# Clinical practice guideline exercise and lifestyle in chronic kidney disease

**DOI:** 10.1186/s12882-021-02618-1

**Published:** 2022-02-22

**Authors:** Luke A. Baker, Daniel S. March, Thomas J. Wilkinson, Roseanne E. Billany, Nicolette C. Bishop, Ellen M. Castle, Joseph Chilcot, Mark D. Davies, Matthew P. M. Graham-Brown, Sharlene A. Greenwood, Naushad A. Junglee, Archontissa M. Kanavaki, Courtney J. Lightfoot, Jamie H. Macdonald, Gabriella M. K. Rossetti, Alice C. Smith, James O. Burton

**Affiliations:** 1 University of Leicester, Leicester, UK; 2 Loughborough University, Loughborough, UK; 3 King’s College Hospital, London, UK; 4 King’s College London, London, UK; 5 Betsi Cadwaladr University Health Board and Bangor University, Bangor, UK; 6 Cardiff and Vale UHB, Cardiff, UK; 7 School of Sport, Health and Exercise Sciences, Bangor University, Bangor, UK; 8 University of Reading, Reading, UK; 9 University of Leicester and Leicester Hospitals NHS Trust, Leicester, UK

## Background

The statement that ‘if exercise were a pill it would be one of the most widely prescribed and cost-effective drugs ever invented’ has been used many times, with many slightly different iterations and with good reason; because the evidence is compelling, and the message is clear that being active provides a foundation for a longer, healthier and happier life.

Although other national and international kidney disease guideline documents include some basic recommendations for physical activity and lifestyle, at the time of publication this is the first document of its kind to set out the evidence for those people living with kidney disease, including those on haemodialysis and with a kidney transplant.

The scope of these guidelines was agreed by a multi-professional group of healthcare experts, experienced in this field, over three separate meetings of the UK Kidney Research Consortium Clinical Study Group for Exercise and Lifestyle. The authors and guideline development group entirely accept that physical activity recommendations comprise the majority of this document; this is intentional to avoid duplicating expert evidence that can be found elsewhere. Throughout, these national and international resources have been signposted, where appropriate.

Systematic literature searches were undertaken to identify all published clinical evidence relevant to the review questions and the exact parameters are outlined below. As well as pragmatic audit measures, we have included ‘Points for implementation’ which we hope will help to translate some of the recommendations into clinical practice in your units.

The group would like to particularly highlight the contributions of Drs Baker, March and Wilkinson who led the evidence reviews for the CKD, haemodialysis and transplantation sections, respectively.

### Non-dialysis CKD section

#### Objective

To perform a search for randomized control clinical trials (RCTs), systematic reviews and meta-analyses that will subsequently inform the writing of the new Renal Association Guidelines for physical activity and lifestyle in the CKD stages 1–5 (non-dialysis) population.

#### Study eligibility and criteria

Publications were considered for inclusion if they were RCTs that involved allocation of participants at an individual or cluster level, or via quasi-randomised method. Systematic reviews alone or systematic reviews with meta-analysis of these trials were also considered for inclusion. Within these criteria, publications were subsequently screened for their target population in order to only include studies which studied the non-dialysis CKD population (stages 1–5) of adult years (> 18 years).

In order to identify those studies related to the guideline topic, studies which researched physical activity, exercise, lifestyle, weight loss or smoking cessation which aimed to discuss or improve outcomes (clinical or patient reported) in non-dialysis CKD patients (stages 1–5) were included. For this purpose, the following definitions were employed:

- Physical activity - habitual activity which includes bodily movement produced regularly by the contraction of skeletal muscles that result in a substantial increase over resting energy expenditure as part of activities of daily living.
- Exercise – activity, which was planned, structured, and repetitive bodily movement.

In topic areas in which there is currently insufficient RCTs available, epidemiological studies were included in the synthesis of studies. Utilising the expertise of the reviewing team, a pragmatic search of current guidelines available was also conducted.

#### Data collection and extraction

Searches of systematic review databases was conducted, Cochrane and PROSPERO. MEDLINE was searched which includes the National Centre for Biotechnology Information (NCBI) PubMed. One author independently reviewed the title of every record retrieved from the electronic search. If the information given in the title suggested that the study might fit the inclusion criteria of the systematic review, the abstract was read. If the title and abstract suggested that the study might fit the inclusion criteria of the systematic review, the full article was retrieved for further assessment. Studies that did not fulfil the selection criteria of the systematic review were eliminated. The section leads then reviewed the retrieved studies to confirm whether they met the inclusion criteria. The list of search terms can be found for each of the search areas within the wider topic area in Additional file 1: Appendix ND-CKD1. Corresponding flow citation charts of the search process can be found in Additional file 1: Appendix ND-CKD2.

Following searches, quantitative data from each review was independently extracted by the one author, which was then reviewed and approved by the section leads with variations resolved by consensus, referring back to the original data. Data was subsequently synthesized narratively in guideline format, with statements regarding the evidence being made and graded using the modified GRADE system [1].

### Haemodialysis section

A systematic review of recent systematic reviews, meta-analyses and randomised controlled trial data pertaining to physical activity and exercise studies for individuals with end-stage kidney disease (ESKD) receiving haemodialysis was conducted to provide an up-to-date evidence base. The methodology and search strategy can be found in Additional file 1: Appendix HD1 and HD2.

### Transplantation section

We first reviewed and summarised current evidence that has investigated epidemiological evidence on either physical activity and exercise levels in Kidney Transplant Recipients (KTRs) and/or the association between physical activity and exercise levels with outcomes. A systematic search of existing systematic and narrative reviews of physical activity and exercise in KTRs was conducted. NCBI MEDLINE (1966-present day) was searched using the following MESH search terms: kidney transplantation; transplant recipients; exercise; exercise therapy. An example of a full search strategy can be found in Additional file 1: Appendix TX1. To gather the most recent evidence available, only reviews published in the last 5 years were sought (2015 to 2020). After full-text review, a total of 14 reviews relating to physical and activity in renal transplant recipients were identified. These reviews were hand-searched, and the authors sought each review for appropriate information, references of studies, and data pertaining to physical activity and exercise levels in KTRs, and the association with outcomes.

Secondly, we conducted, where appropriate, a pragmatic hand-search of all current guidelines and position statements pertinent to lifestyle, physical activity, and exercise levels in KTRs. Finally, we conducted a systematic search and meta-analysis of randomized clinical trials studying the effect of received a physical activity or exercise intervention, either supervised or unsupervised, on outcomes in patients with (or awaiting) a kidney transplant. The following electronic databases were searched from their date of establishment to January 2020: National Centre for Biotechnology Information (NCBI) PubMed (which includes the Medical Literature Analysis and Retrieval System Online (MEDLINE)), and the Cochrane Central Register of Controlled Trials (CENTRAL) (includes Excerpta Medica database (EMBASE), and the WHO International Clinical Trials Registry Platform (ICTRP)). The following MESH search terms were used to search all databases: kidney transplantation; transplant recipients; exercise; exercise therapy; randomised controlled trial. Full search strategies can be found in Additional file 1: Appendix TX2. A flow of information through the different phases of the search can be found in the figure in Additional file 1: Appendix TX3. Complete tables (Additional file 1: Appendix TX4), forest plots (Additional file 1: Appendix TX5), risk of bias summary (Additional file 1: Appendix TX6), Leave-one-out’ sensitivity analysis (Additional file 1: Appendix TX7), and funnel plots (Additional file 1: Appendix TX8) relevant to this meta-analysis can be found in the appendices. The strengths of the recommendations and the level of supporting evidence are coded as previously using the Modified GRADE system.

## Summary of recommendations

### Non-dialysis CKD (stages 1–5)

#### Physical activity and exercise

1.1 We recommend physical activity should be encouraged in the non-dialysis CKD population without contraindications and with stable, controlled comorbidities (1B).
1.2 We recommend non-dialysis CKD patients follow the UK Chief Medical Officers’ Physical Activity Guidelines (2019), slightly adapted for this population (1B):
  Non-dialysis CKD patients should participate in daily physical activity. Some physical activity is better than none.
  Non-dialysis CKD patients should maintain or improve their physical function by undertaking activities aimed at improving or maintaining muscle strength, balance and flexibility on at least 2 days a week.
  Non-dialysis CKD patients should aim to accumulate 150 min of moderate intensity aerobic activity per week, building up gradually from current levels. Those who are already regularly active can achieve these benefits through 75 min of vigorous intensity activity per week, or a combination of moderate and vigorous activity.
  Non-dialysis CKD patients should break up prolonged periods of being sedentary with light activity when physically possible, or at least with standing.
1.3 We recommend that increasing physical activity or exercise levels in non-dialysis CKD patients will contribute to the following:
  Improvements in blood pressure (1B).
  Improvements in physical function and capacity (1B).
  Improvements in functional limitations (1C).
  Improvements in health-related quality of life (1C).
1.4 We suggest that exercise may improve mental well-being, e.g. symptoms of depression and anxiety (2C).
1.5 We recommend that a prescribed combination of aerobic and muscle strengthening should be utilised to improve muscle function (1C).

#### Weight management

1.6 We recommend that anthropometrics should be measured and monitored (self-monitored if necessary) at regular intervals in individuals with non-dialysis CKD (1B).
1.7 We recommend that multi-professional weight management services should be available to all non-dialysis CKD patients, with referral made to tier 3 services (in-line with regional referral pathways) where appropriate (e.g. when notable changes to anthropometrics are observed) (2D).

#### Other lifestyle considerations (smoking, alcohol intake, drug use)

1.8 We recommend that individuals diagnosed with non-dialysis CKD (stages 1–4) stop smoking (1A).
1.9 We recommend alcohol consumption should be within national guidelines (1B).
1.10 We recommend that individuals avoid all recreational drug use (1B).

### Haemodialysis

#### Physical activity and exercise

2.1 We recommend that physical activity and exercise should be encouraged in the haemodialysis population where there are no contraindications (1C)
2.2 We recommend that haemodialysis patients should aim for 150 min of moderate intensity activity a week (or 75 min of vigorous activity) or a mixture of both as per the UK Chief Medical Officers’ Guideline. This may include a combination of exercise outside of dialysis (interdialytic) or exercise during dialysis (intradialytic) (1B).
  We suggest that sufficient physical activity may reduce risk of cardiovascular related and all-cause mortality in the haemodialysis population (1C).
  We suggest that increased physical activity or exercise may have favourable effects on blood pressure (2C).
2.3 Exercise during haemodialysis (intradialytic exercise) is safe with no contraindications; we therefore recommend that it should be available in all units
  To improve cardiovascular health and physical function (1B).
  To improve muscular strength (2C).
  Reduce hospitalisations (2C)
  To improve blood pressure control (2C).
  To improve lipid profiles (2D)
  To improve dialysis efficiency (2D).
2.4 We suggest that programmes for increasing physical activity and exercise are supervised and led by individuals qualified to deliver exercise and/or rehabilitation programmes in populations with chronic disease (2D).
2.5 We recommend that individual participant and staff barriers need to be addressed to optimise programme participation and adherence (1C).

#### Weight management

2.6 We recommend that regular anthropometric measurements should be taken to assess changes in body composition [1B]
2.7 We recommend that all individuals receiving haemodialysis maintain a BMI of between 20 and 30 kg/m2 (1C).
2.8 We recommend that a multi professional approach should be taken to weight management. This should include the evaluation of nutritional needs along with comorbid conditions, and the promotion of physical activity and exercise supported by behaviour change techniques (2C).
2.9 We suggest that bariatric surgery is safe and may be considered for those individuals wishing to receive a transplant for whom current BMI prevents this (2C).

#### Other lifestyle considerations (smoking, alcohol intake, drug use)

2.10 We recommend that individuals receiving haemodialysis stop smoking (1A).
2.11 We recommend alcohol consumption should be within national guidelines (1B).
2.12 We recommend that individuals receiving haemodialysis avoid all recreational drug use (1B).

### Transplantation

#### Physical activity and exercise

3.1 We recommend that general physical activity should be encouraged in KTRs without contraindications [1B]
3.2 We suggest sufficient physical activity, pre- and post-transplant, can reduce all-cause and cardiovascular mortality [2C]
3.3 We recommend KTRs aim for 150 min of moderate to vigorous physical activity a week (or 75 min vigorous physical activity) as per the UK Chief Medical Officers’ Guideline [1C]
3.4 We suggest individual barriers and activators to physical activity need to be identified and addressed to optimise programme uptake and adherence [2C]
3.5 We recommend that structured exercise be considered as a method of enhancing cardiorespiratory fitness [1B]
3.6 We recommend that structured exercise be considered as a method of enhancing muscular strength and physical function [1C]
3.7 We suggest that structured exercise be considered as a method of improving health-related quality of life and increasing HDL levels [2C]
3.8 Structured exercise alone is not sufficient to attenuate increases in body mass following transplantation; we therefore suggest a multi-professional approach to appropriate weight-management strategies [2B]
3.9 We suggest that structured exercise should be performed at least 3x/week in KTRs without contraindications [1C]
3.10 We suggest that KTRs without contraindications undertake both aerobic and resistance exercise to maximise the effects on exercise capacity and muscle function [1B]
3.11 We suggest that a structured exercise routine be devised (and supervised if possible) by appropriately trained staff [2B]
3.12 We suggest exercise programmes should be individualised based on underlying patient goals/expectations, pathophysiology, level of experience, and graft status [2C]

##### Prehabilitation for transplantation

3.13 We suggest that exercise interventions prior to surgery (prehabilitation) may help increase pre-transplant physical activity levels and aid recovery post-transplant [2C]

##### Immediate post-transplantation period

3.14 We suggest that exercise interventions consisting of intensive physiotherapy and movement encouragement administered immediately post-transplantation i.e. < 1–2 days is not beneficial in increasing recovery or attenuating declines in physical function. However, mobility should be encouraged as per standard care [2C]

##### Safety and contraindications

3.15 We suggest that KTRs avoid traumatic damage to the transplanted kidney and participation in contact sports (e.g., rugby, American football, martial arts, ice hockey, boxing) and/or prolonged extreme exercise (e.g., marathons, Ironman triathlons) must be considered carefully [2C]
3.16 We suggest that KTRs avoid the use of sport-enhancing dietary supplements given the largely unknown potential adverse effects on immune function and potential for unregulated components [2C].

#### Weight management

3.17 We recommend that regular anthropometric measurements should be taken to assess changes in body composition [1B]
3.18 We recommend candidates and KTRs have their body mass (and body mass index, BMI) accurately examined by a healthcare professional at the time of evaluation and while on the waiting list [1B]
3.19 We recommend not excluding candidates based on BMI alone [1B]
3.20 We recommend that potential recipients, not on dialysis, with a BMI > 35 kg/m2 should be actively supported to lose weight via appropriate interventions [1C]
3.21 We recommend that multi-professional weight management services should be available to all KTRs [1C]
3.22 We recommend that post-transplantation an ideal weight should be targeted BMI ≤25 kg/m2) [1B]
3.23 We suggest bariatric surgery can be used to reduce BMI in those with morbid obesity (i.e., >BMI 40 kg/m2)’ [2B]

#### Other lifestyle considerations (smoking, alcohol intake, drug use)

3.24 We recommend that smoking should be strongly discouraged in transplant recipients [1A]
3.25 We suggest alcohol consumption should be within national guidelines [1B]
3.26 We suggest that KTRs avoid all recreational drug use [1B]

## Non-dialysis CKD (stages 1–5)

### Introduction

Regular physical activity and exercise is associated with numerous physical and mental health benefits in the general population [2]. All-cause mortality is delayed through the regular undertaking of physical activity whilst also leading to reductions in the risk of developing cardiovascular risk factors such as elevations in blood pressure. Improving physical activity levels in line with current recommendations leads to reduced risk of the development of diabetes, stroke and some cancers (e.g. colon and breast cancer) [2]. With the benefits in the general population clear, research has focussed on understanding whether similar benefits are noted in those with non-dialysis Chronic Kidney Disease (CKD).

CKD is a long-term condition with a significant proportion of those affected never reaching end-stage renal disease (ESRD) where dialysis or renal replacement therapy is required, and therefore remains in stages 1–4 of the disease. This population suffer from a high symptom burden, impaired physical function and reduced physical activity levels [3]. Such factors have been linked to reduced quality of life and more recently associated with elevated levels of all-cause mortality, reduced risk of cardiovascular mortality and increased risk of rapid decline in renal functions [4]. As such, intervening through the means of physical activity and exercise in this population may provide an opportunity for the alleviation of symptoms in the short-term improving quality of life, but also benefit patient outcome through their disease progression and mortality risk [5].

Appropriate self-management and a healthy lifestyle are recommended to patients with CKD stages 1–5 with the aim of minimising symptom burden and reducing the risk of disease progression and cardiovascular events. A core component of generalised lifestyle advice is the concept of physical activity. Physical inactivity is one of the major risk factors for mortality in the general population [6] and multiple studies have shown that in the general population, physical activity is associated with a less deleterious CVD risk-factor profile leading to fewer adverse cardiovascular outcomes [7, 8] alongside increased quality of life. Increasing exercise and physical activity levels poses a viable option for addressing many of the underlying factors which affect the non-dialysis CKD population. Thus, these guidelines refer to the relationship between physical activity, exercise and the related lifestyle factors of smoking, alcohol and drugs on clinical and patient-reported outcomes in adult patients with non-dialysis CKD stages 1–5.

### Physical activity and exercise

The following section provides a synthesis of the current evidence in order to make informed recommendations in regard to the effect of physical activity and exercise in non-dialysis CKD patients (stages 1–5). Additionally, this includes extensive rationale alongside audit measures and tips for implementation in this population.

### Recommendations

1.1 We recommend physical activity should be encouraged in the non-dialysis CKD population without contraindications and with stable, controlled comorbidities (1B).
1.2 We recommend non-dialysis CKD patients follow the UK Chief Medical Officers’ Physical Activity Guidelines (2019), slightly adapted for this population (1B):
  Non-dialysis CKD patients should participate in daily physical activity. Some physical activity is better than none.
  Non-dialysis CKD patients should maintain or improve their physical function by undertaking activities aimed at improving or maintaining muscle strength, balance and flexibility on at least 2 days a week.
  Non-dialysis CKD patients should aim to accumulate 150 min of moderate intensity aerobic activity per week, building up gradually from current levels. Those who are already regularly active can achieve these benefits through 75 min of vigorous intensity activity per week, or a combination of moderate and vigorous activity.
  Non-dialysis CKD patients should break up prolonged periods of being sedentary with light activity when physically possible, or at least with standing.
1.3 We recommend that increasing physical activity or exercise levels in non-dialysis CKD patients will contribute to the following:
  Improvements in blood pressure (1B).
  Improvements in physical function and capacity (1B).
  Improvements in functional limitations (1C).
  Improvements in health-related quality of life (1C).
1.4 We suggest that exercise may improve mental well-being, e.g. symptoms of depression and anxiety (2C).
1.5 We recommend that a prescribed combination of aerobic and muscle strengthening should be utilised to improve muscle function (1C).

### Audit measures

1. Physical activity should be monitored through the use of a validated physical activity questionnaire: such as the General Practice Physical Activity Questionnaire (GPPAQ) – a NICE recommended survey to help identify those inactive and in need of support or the Physical Activity Vital Sign (PAVS) (endorsed by the American College of Sports Medicine (www.acsm.org) Exercise is Medicine®).

### Points for implementation

- Health care professionals in renal settings should be aware of local exercise prescription policies and other localised physical activity referral programmes to be able to refer patients to these services.
- Regular conversations about exercise should be held with patients during their clinical visits to raise awareness of the benefits of exercise.
- Non-dialysis CKD patients should aim to minimise the amount of time spent being sedentary, and when physically possible should break up long periods of inactivity with at least light physical activity.
- Physical activity can comprise of general work or leisure-time physical activities, structured exercise, or sport, as appropriate.
- When possible, exercise should be supervised for greatest compliance and efficacy by an appropriately trained individual (e.g. physiotherapist, sport scientist, cardiac rehabilitation specialist or an assistant physiotherapist/dietitian/nurse with additional training from one of the former groups) particularly in patients with complex medical comorbidities. However, lack of access to trained individuals should not prevent facilitating patients to increase their physical activity.
- For guidance around training and development of the multi-professional team to implement such guidance, see upcoming Global Renal Exercise (GREX) training and development programme. (https://grexercise.kch.illinois.edu).

### Rationale

#### Mortality and disease progression

Research to date regarding the beneficial effects between physical activity/exercise and mortality, hospitalisations and disease progression is limited in its consistency. As such, we are currently unable to make any recommendations related to these outcome measures.

Much of the research in relation to these outcome measures in ND-CKD is derived from observational study designs, using retrospective analysis and self-reported physical activity levels which have innate scientific limitations. In regard to mortality, one recent meta-analysis and systematic review concluded that the use of self-management interventions (which included exercise) showed no significant difference in risk of all-cause mortality [9]. One further study has since attempted to further look at physical activity and associations with all-cause mortality. Chen and colleagues reported that as part of the MDRD study in a cohort of 811 patients, no associations were noted with different types of self-reported physical activity and mortality [10]. A recent study has recently reported an association between quadriceps cross sectional area and all-cause mortality; however, these associations were non-significant once adjusted, indicating quadriceps volume is not a driving factor in regard to all-cause mortality in this population [5]. There is also limited available evidence regarding a link between physical activity and exercise and subsequent hospitalisations. One study has reported a non-significant interaction between an aerobic training group and usual care control group in ambulatory heart failure patients and CKD [11].

When discussing the relationship between physical activity and exercise with disease progression, it is important to separate findings relating to different outcome measures. A recent meta-analysis including 31 trials which undertook aerobic exercise programmes (totalling *n* = 1305) concluded there was no difference in serum creatinine between control and training groups post intervention period [12]. However, a recent report did conclude that a self-management intervention (which included an exercise component) was able to show improvements in serum creatinine after a 3-month period [13]. As the exercise here was part of a wider intervention it is hard to infer cause and effect; however further studies are warranted to follow up on such findings. As the most common indicator used for disease progression in CKD populations, the effect of exercise and physical activity on eGFR has rightly been investigated. The current research available has been summarised in 3 meta-analyses which concluded conflicted findings. Two of these analyses reported that aerobic exercise programmes show no discernible effects upon eGFR, with most of the included studies focussed around self-management interventions which contained an exercise element [9, 14]. The other meta-analysis reported that, with the inclusion of 11 RCTs (totalling *n* = 362 patients) with an average of 35-weeks of aerobic exercise, eGFR was increased (2.16 ml/min [0.18;4.13] [15]. However, the authors did concede that the effects were small, and the data was limited due to many of the included studies being low to moderate quality. As such we believe that though positive effects have been reported in regard to exercise and eGFR, current evidence does not warrant recommendations in the non-dialysis CKD population. A final measure of disease progression which has been studied is the role of exercise and physical activity in moderating albuminuria/proteinuria. Recent research in this area has been summarised, which included those who studied the effect of self-management interventions (which included exercise) on albuminuria [9]. Conclusions suggested that self-management interventions were associated with lower 24 h protein excretion. Two studies, which were not included in this analysis, both looked at an exercise-only intervention and reported no significant changes in either a non-dialysis CKD-obese population [16] or after a 24-week exercise intervention in non-dialysis CKD patients only [17]. Overall conclusions from the research base investigating exercise and physical activity on disease progression markers is that currently, the work presented shows no basis for specific recommendations.

#### Physical activity

It has long been hypothesised that participation in physical activity may have beneficial effects on long term outcomes in people living with chronic kidney disease. However, in contrast to the general population, there is limited evidence in ND-CKD of such beneficial effects due to the difficulties in completing studies within this population. Nevertheless, it is reasonable to assume that benefits of physical activity will also be realised in ND-CKD, and importantly reviews [18] completed in ND-CKD suggest there is no reason to believe that risks of physical activity participation outweigh potential benefits in this population. Therefore, for pragmatic reasons we have decided to adapt and present the UK Chief Medical Officers’ Physical Activity Guidelines (2019) which are based on extensive review and have been recently updated. As many patients with ND-CKD are older adults, and frailty is common in ND-CKD, the Older Adults guideline was used as a cautious starting point. Use of the Older Adults guideline also avoids recommendation of high intensity interval training (HIIT), which although included in the adult (19 to 64 years) guideline, is not deemed to have a high enough benefit to risk ratio in ND-CKD patients of any age. We have also decided to remove the recommendation to complete weight bearing activities which create an impact through the body to maintain bone health, as it is not clear if this is safe or effective in patients ND-CKD patients with complicated bone health. As a loss of muscle strength is the primary limiting factor for functional independence [19], and falls risk is high in ND-CKD patients, multi-component strength and balance activities, including flexibility, are recommended.

As per the UK Chief Medical Officers’ Physical Activity Guidelines (2019), we also suggest that all individuals work towards achieving the physical activity levels included in these guidelines but there are no absolute thresholds: benefits are likely to be realised at levels below the guideline. Light intensity physical activity is associated with a range of health and social benefits in the general population and there is no reason to assume it will not also be beneficial in people living with ND-CKD. Note there is no minimum bout length of 10 min of physical activity (as was previously recommended) – even sporadic accumulated activity is likely to be beneficial [20]. Alternative ways of recording physical activity, such as with pedometers, may be helpful to some adults to encourage and record habitual physical activity behaviour. Achieving 4500 steps per day may convey quality of life benefits; achieving 10,000 steps per day may support maintenance of body weight. Many ND-CKD patients are sedentary, which is associated with poor health and functional capacity [21, 22]. There is emerging evidence that light physical activity and even short periods of standing can benefit health and physical function and are thus recommended.

#### Physical function, physical capacity and muscle mass

It is well documented that patients with CKD suffer from a reduced exercise capacity and poor levels of physical functioning which leads to a cyclic reduction in physical activity levels and consequently deconditioning [23]. Unsurprisingly this spiral of inactivity has been shown to impact the quality of life of this patient population as well as contributing to poorer outcomes (morbidity and mortality) [4, 5]. Research to date has focussed on the effect of physical activity and exercise interventions on muscle mass, muscle strength and physical capacity. Relating to muscle mass, to date of the 14 studies which sought to investigate the effect of differing exercise interventions, 6 reported a positive effect on muscle mass [24–26] or muscle volume [27] with a mix of high and low risk of bias, with 10 of these studies being RCTs in design. Of the RCTs, 8 studies contained an aerobic training element with 38% (3/8 studies) reporting beneficial effects of aerobic training on muscle mass or a surrogate marker of muscle mass (ie. fat free mass %). One study contained a resistance exercise only element [28] showing a positive effect on lean mass (kg), another showed a positive effect of combined aerobic and resistance training elements in comparison to an aerobic only intervention on quadriceps volume (cm^3^) [27], both of which displayed a low risk of bias. The final study showed beneficial effects of a HIIT based program of exercise on muscle fibre area, although this study had a high risk of bias [29].

The effects of physical activity and exercise on muscle strength seem to be relatively consistent to this point leading to the above recommendation, though there is variation in assessments used to quantify muscle strength. To date, two studies assessed as low risk of bias have reported increases in 1-repetition maximum values in response to a 12-week training programme containing a resistance component [27, 29]. Further to this, 3 RCTs have reported increases in quadricep strength [30–32] all of which contained a resistance exercise element ranging from 8 weeks to 12 months in length. One study to date has shown the effects of a combined exercise programme on hand grip strength, which is of particular interest as this is the assessment of choice for the diagnosis of sarcopenia. Hiraki and colleagues [31] showed significant improvement in hand grip strength in response to a home based combined intervention programme with increases of 17%, though it should be noted that the current study was a small sample size (*n* = 36) and further studies should aim to confirm such findings in this important measurement.

A larger body of research has investigated the effect of physical activity and exercise interventions on physical capacity and exercise tolerance in non-dialysis CKD patients. Currently there are 14 studies published investigating physical capacity, 13 of which are RCTs and one of which is observational [33] which have been recently synthesised in two meta-analyses. Both recent analyses reported a positive effect of exercise, which included both aerobic and aerobic plus resistance-based elements [34, 35]. The most recent of these meta-analyses concluded that across the 6 RCTs included [16, 24, 36–38], exercise interventions lead to positive effects in the 6-min walk test (6MWT), across the *n* = 212 non-dialysis CKD patients included. The same analysis reported a significant effect of exercise interventions on the number of bicep curl repetitions performed by patients, which was concluded from two studies conducted by the same group [24, 36]. These findings were concurrent with another recent meta-analysis which concluded a positive effect of exercise on the 6MWT which included five recent RCTs [35]. Further to recent meta-analyses, Watson and colleagues [27] reported a significant increase in the intermittent shuttle walk test (ISWT) post 12 weeks of either structured aerobic or combined exercise, which is one of the largest single RCTs to date to investigate such an intervention on physical function in non-dialysis CKD patients. Exercise tolerance has predominantly been measured through the use of VO_2_ peak, which has been reported at length in the non-dialysis population. Research has been synthesised in four recent meta-analyses over the past 2 years, all of which conclude a beneficial effect of an extended exercise intervention (ranging from 12 to 52 weeks) on VO2 peak in non-dialysis CKD patients [34, 35, 39, 40]. The most recent of these [40] reported that in comparison to standard care aerobic training improved VO_2_ peak by 2.39 ml/kg/min (CI 0.99; 3.79). This is considered clinically relevant by the authors based upon the minimal clinically important difference of 2.00 ml/kg/min.

#### Metabolic risk factors

##### Blood pressure

There are four recent high-quality systematic reviews with meta-analyses of RCTs on the effects of exercise on blood pressure. Zhang and colleagues [41] included 9 studies including 14 different time-based analyses with a cumulative patient population of *n* = 463. The included trials spanned from 16 to 52 weeks in length with assessment time points across these time periods, with all studies containing a supervised element with the exception of one [24] which used a home-based exercise model. Upon meta-analysis a reduction of systolic blood pressure (SBP) was noted (5.61 mmHg; *p* = 0.001), and in further sub-group analysis reductions in SBP were noted regardless of the exercise training. Similar reductions were noted in diastolic blood pressure (DBP) (2.87 mmHg; *p* < 0.01). Thompson and colleagues [42] conducted a meta-analysis of 8 studies with the inclusion of 335 non-dialysis CKD patients. Though all studies were at a high risk of bias, they found significant reductions in SBP after 12–16 and 24–36 weeks of exercise (4.98 mmHg and 10.94mmHG respectively), but no difference at 48–52 weeks. The other recent meta-analysis [15] showed within-group improvements in SBP. There were no between-group differences (between exercise groups and standard care) at the end of the intervention periods, but that analysis was based on a limited number of studies. There has also been a handful of investigations published outside of the currently available meta-analyses. An RCT looking at the effects of a HIIT training programme compared to moderate-intensity continuous training (MICT) showed reductions in both SBP and DBP (9.8 mmHg and 11.00 mmHg respectively) across the groups but no difference between the training modalities [43]. Two further studies both conducted a 12-week exercise intervention [27, 44] containing an aerobic or combined aerobic and resistance-based elements, neither of which reported a difference in blood pressure, suggesting a longer time course is required. It was also noted that the majority of studies to date have failed to control for changes in medication management, which is not surprising in interventions of this length, often making changes in blood pressure hard to associate with exercise directly in many cases, but also potentially diluting the effect of exercise, if there were concomitant antihypertensive dose reductions during the study period [40]. However, despite this limitation we still believe there is sufficient evidence to warrant the current recommendation that exercise can improve blood pressure, though future work should attempt to consider and account for the clear effects that changes in medication management will have on blood pressure in such interventions.

##### Blood lipids

The effects of physical activity and exercise on blood lipids has been examined in 5 systematic reviews. Of these, two also included ESRD patients and showed no significant changes in total cholesterol, high-density lipoprotein cholesterol (HDL-C), low-density lipoprotein cholesterol (LDL-C) or triglycerides [45, 46]. One systematic review [41] did infer beneficial effect on triglyceride levels in the short term (< 3 months); however, the benefit was not apparent in analysis of longer duration intervention (up to 12 months) [41]. Pei and colleagues [12] reported a small but significant increase in HDL-C (3.54 mg/dL) from six randomised controlled trials (RCTs) which looked at exercise interventions in non-dialysis CKD, but no further benefits were noted. The above findings were recently confirmed in the most recent systematic review and meta-analysis which concluded that current available evidence suggests that exercise interventions have little to no effect on blood lipid levels in non-dialysis CKD patient populations [39]. Our own systematic review found an additional two RCTs which studied the effect to exercise therapy on blood lipids [47, 48]; they both found no benefit of combined exercise training on any component of the blood lipid profile. Overall, our conclusion of the current research base is that there is no beneficial effect of exercise in total cholesterol, HDL-C, LDL-C or triglycerides. Thus, we are currently unable to recommend exercise as an intervention to improve blood lipid levels or composition.

##### Glycaemic control

There are currently no published systematic reviews which have examined the effects of exercise or physical activity interventions on fasting blood sugar levels or HbA1C in non-dialysis CKD patients. One systematic review [49] reports that aerobic and resistance exercise can reduce mean HbA1C, though the studies included were not designed for this purpose and included patients with CKD alongside other diabetic populations. Following a review of the current RCTs available, seven were noted to report HbA1C as an outcome. These RCTs reported a variety of exercise interventions, including home-based [50], aerobic [17, 51], and exercise combined with other lifestyle interventions [16, 52, 53]. Only one study, using aerobic exercise either supervised or at home for 24 weeks, showed beneficial effects on HbA1c [36]. One other study has reported a benefit on glycaemic control: Barcellos and colleagues [48] did report reduced fasting glucose levels in response to an exercise intervention 16 weeks in length (reduced by 11.3 mg/dL). With this in mind, based upon the current research we are unable to make recommendations for the use of exercise to induce beneficial changes in glycaemic control, though further research is required which is specifically designed to address this question in non-dialysis CKD patients.

#### Inflammation

There are currently no published systematic reviews or meta-analyses in regard to the effects of exercise on inflammation. A review of the current research base identified 15 studies that measured at least one inflammatory factor: CRP (*n* = 13); interlukin-6 (*n* = 4), albumin (*n* = 3); interlukin-10 (*n* = 1); transferrin (*n* = 1). Twelve of these studies saw no effect of exercise interventions on systemic markers of inflammation [16, 17, 24, 51, 52, 54–60]. Inflammatory reductions were noted in IL-6 and CRP after a 12-week resistance exercise intervention [29]; in this study both control and exercise groups were advised to follow low-protein diets. This is the only study which has looked at the effect of resistance-only exercise on inflammatory indicators in non-dialysis CKD patients. Beneficial effects of exercise including reductions in IL-6 after 4 months’ aerobic exercise with or without calorie restriction [61], and attenuations of CRP elevations with exercise in comparison to increased levels noted in the non-exercise control group after 16 weeks combined aerobic and resistance training have been shown in other studies [48]. Overall the current evidence for an effect on inflammation is limited. With this in mind we are currently unable to make specific recommendations in regard to the effect of exercise on inflammation, though resistance exercise may offer a potential avenue in the future if the robust research required in this area is undertaken.

#### Cognitive function

The physiological effects of exercise are commonly described and reported at length in the general population and more recently in CKD populations. However, the potential of exercise to affect cognition is still vastly under researched, particularly in chronic disease populations such as CKD. To date there are no experimental studies which have looked at the effects of exercise on a direct measure of cognitive function in the non-dialysis CKD population. One recent observational study reported a relationship between physical function (walking gait speed) and cognitive function, measured using the Japanese version of the MoCA-J [62]. However, without a direct measure of cognitive function and a manipulation of physical activity levels, causality is unable to be identified. The only other study within the non-dialysis CKD population looked at the effect of a three-month home-based aerobic exercise programme on the KDQOL-SF questionnaire [59]. Findings reported a significant improvement in cognitive function through this measure, though again this is not a direct measure of cognitive function. With this in mind we are unable to make a recommendation regarding the use of exercise to improve cognitive function, however we wish to emphasise the importance of attenuating the decline of cognitive function in the non-dialysis population and highlight this as a particular area of research which needs addressing.

#### Health related quality of life

A significant amount of research has sought to determine whether exercise is able to increase health related quality of life (QoL) in ND-CKD patients. On review of the currently available literature, we recommend that increasing levels of physical activity or the undertaking of regular exercise can indeed increase QoL. The current literature has been summarised in three recent systematic reviews which has summarised recent RCT trials investigating the effect of increasing physical activity levels on QoL. The most recent of these [34] assessed the findings of six recent RCTs, concluding a significant positive effect of an exercise interventions, predominantly aerobic based, on QoL measures such as the SF-36 (*p* = 0.02) and the KDQOL-36 (*p* = 0.02). Authors reported differences of 5.7 points in the SF-36, which has reported to be clinically important in ND-CKD previously [63]. A separate group of authors also conducted a meta-analysis on this topic [35] drawing similar conclusions. The authors conducted a meta-analysis of six studies which measured the SF-36 and analysed the effect of exercise on each domain of this measuring tool. Positive effects of exercise interventions were noted on physical role, physical functioning, vitality and bodily pain. With regard to particular exercise modalities in order to improve QoL, a recent meta-analysis investigated the effect of combined aerobic and resistance exercise [39]. On analysis of the two studies included, the authors reported no significant effect of combined exercise training on physical or mental QoL scores. The authors concluded that further studies into differing exercise modalities are required as the research base is currently limited. As such we are currently unable to make recommendations regarding specific exercise modalities when attempting to improve QoL in ND-CKD patients.

### Weight management

The following section provides a synthesis of the current evidence in order to make informed recommendations in regard to the effect of physical activity and exercise in non-dialysis CKD patients (stages 1–5) with relationship to weight management. Additionally, this includes extensive rational alongside audit measures and evidence for safety in this population.

### Recommendations

1.6 We recommend that anthropometrics should be measured and monitored (self-monitored if necessary) at regular intervals in individuals with non-dialysis CKD (1B).
1.7 We recommend that multi-professional weight management services should be available to all non-dialysis CKD patients, with referral made to tier 3 services (in-line with regional referral pathways) where appropriate (e.g. when notable changes to anthropometrics are observed) (2D).

### Audit measures

1. Anthropometrics should be recorded and monitored during clinical visits. (*For guidance on which anthropometric measures should be utilised, see the indicated link in the rationale)

### Rationale

Obesity is a major risk factor for cardiovascular disease and death in the general population [64]. Data from observational based studies have shown repeatedly that obesity is an independent risk factor of CKD onset. In established CKD patients, both sarcopenia and obesity has been shown to increase mortality risk, and increase likelihood of progression to ESRD [65]. Unlike patients who are receiving dialysis or are leading up to renal replacement therapy, the non-dialysis CKD population requirements are based upon the principle of maintaining a ‘healthy weight’ and the prevention or attenuation of obesity as opposed to needing to account for the relevance of adiposity for surgery or fluid replacement whilst on dialysis. In line with this premise, current NICE guidelines stipulate that the general population should maintain a BMI of between 18.5–26 kg/m^2^ (for Caucasians) and we see no evidence from the current wealth of research for this to differ for the non-dialysis CKD population.

The first aspect to address is the current advised methods of measuring and tracking body composition. The current KDOQI 2020 guidelines [66] infer that Body Mass Index (BMI) is not an ideal marker as it cannot differentiate between increases in adiposity or muscularity, and may thus not identify patients who have sarcopenic obesity (a loss of muscle mass and a concomitant increase in fat mass), or depict the level of visceral fat, which can have negative metabolic effects. However, the authors also state that the use Body Mass Index (BMI) to follow trends over time doesn’t have any potential risk or harm in its usage in the non-dialysis CKD population. Further to this, the authors recommend in ND-CKD populations that when available skinfold thickness, BIS or DXA methods should be used to assess body compositions changes over time, in the absence of oedema (Recommendation 1.1.12; KDOQI 2020). As such, regarding assessment in body composition we refer you to this recent set of guidelines and echo their expert opinion on the topic. The full text can be found at the following link: *https://www.ajkd.org/article/S0272-6386(20)30726-5/fulltext.

With BMI being the primary measure of body composition used in clinical practice, it is important to understand its relationship with CKD onset and mortality risk. A recent systematic review and meta-analysis [67] is the most recent to synthesis the current literature, in which the authors reanalysed the findings of 10 studies with a total samples size of *n* = 484,906 for the systematic review, with 4 of these carried forward for the meta-analyses. These authors concluded that using the WHO designations of BMI stages, in ND-CKD patients stages 3–5 being underweight was associated with a higher risk of death and being overweight or obese class 1 was associated with lower risk of death. This relationship is known as the ‘obesity paradox’ and describes how ND-CKD patients who are either ‘underweight’ or ‘overweight’ are at greater risk of cardiovascular mortality. The U shaped relationship highlights that both ‘underweight’ and ‘overweight’ ND-CKD patients need identification and management.

The modification of body composition is of great interest and we wish to highlight the importance of nutritional and dietary modifications to achieve such changes, and state that exercise interventions should be used in conjunction and not in replacement of dietary modifications. For guidelines in regard to the manipulation of diet, we recommend referring to the recently updated guidance below, and review article with particular focus on protein intake for this patient population: https://kdigo.org/guidelines/diabetes-ckd/

https://www.cochranelibrary.com/cdsr/doi/10.1002/14651858.CD001892.pub5/information.

Aside from dietary manipulation and management, exercise and physical activity provide an opportunity to manipulate weight and body composition. Research to date has focused on the effect of physical activity and exercise on BMI and body weight, as well as fat mass. However, based upon the current evidence available, we are unable to make recommendations of exercise as a tool to modify body composition in the ND-CKD population. Regarding BMI and body weight, there are currently 12 RCTs which have sought to investigate this. Of these studies, only four have reported that exercise interventions lead to significant improvements (i.e. reductions) in body mass or BMI [24, 68–70]. It should be noted that one of these studies reported contradictory findings in BMI and body mass as well as being at a high risk of bias [69]. Regarding exercise modalities, 8 studies contained aerobic components with only 3 of these 8 showing beneficial effects, portraying a weak evidence base for the prescription of aerobic training to obtain changes in BMI or body mass in the non-dialysis CKD population. In regard to resistance exercise alone, one study reported there was no change in BMI [28]. In regard to interventions containing both aerobic and resistance elements combined, two studies reported BMI [68, 71]. Unfortunately, the latter was a feasibility study without sufficient power to report findings, however Howden and colleagues observed a significant reduction in BMI (0.6; *p* ≤ 0.01) in comparison to the baseline values following a 12-month training programme. With this in mind we are unable to currently recommend a specific exercise modality for the management of BMI or body mass, however, further studies should be carried out to determine the potential for the utilisation of combined exercise programmes for this purpose.

Regarding the modification of fat mass, the current evidence base is unable to support any recommendation for the use of exercise as an intervention. There are currently 11 studies which have looked to investigate the effect of exercise on fat mass. Four of these are RCTs and have reported greater levels of physical activity are associated with significant improvements (i.e. reductions) in fat mass [28, 72], body fat percentage [73] or trunk fat mass [24]. The other 7 studies showed no benefit on whole body fat mass [25, 36, 69] or body fat percentage [24, 58, 61, 69]. Interestingly, all studies reporting no effects were conducted using aerobic training methodologies. Just one study has investigated the effect of resistance exercise [28] and reported a reduction in fat mass after a 12-month training programme (− 1.0 kg, *p* = 0.03). However, these effects were also replicated in the balance training control group (− 1.3 kg, *p* = 0.04), suggesting that further research in required in this exercise modality prior to strong recommendations being made. To conclude, we believe that research is warranted to further describe the potential benefits of combined resistance and aerobic exercise in the mediation of body composition and weight management, but these investigations must account for or be conducted in tandem with nutritional interventions and guidelines to portray findings which and transferable to the ND-CKD population to support future recommendations.

### Other lifestyle considerations (smoking, alcohol intake, drug use)

#### Recommendations

1.8 We recommend that individuals diagnosed with non-dialysis CKD (stages 1–4) stop smoking (1A).
1.9 We recommend alcohol consumption should be within national guidelines (1B).
1.10 We recommend that individuals avoid all recreational drug use (1B).

### Audit measures

1. Track the proportion of non-dialysis CKD patients who smoke.
2. Track the number of non-dialysis CKD patients who are referred to the smoking cessation support programme.
3. Track the proportion of non-dialysis CKD patients who suffer from excess alcohol intake.
4. Track the number of non-dialysis patients who are referred to ‘Drink Aware’ support programmes.

### Rationale

There is no doubt that smoking induced disease states are one of the leading causes of mortality in many countries. The Multiple Risk Factor Intervention Trial (MRFIT) was able to document that smoking was significantly associated with increased risk of ESKD [74]. Multiple studies since have shown correlations between smoking and renal dysfunction, with the PREVEND study suggesting that smoking > 20 cigarettes per day lead to elevated risk for high urine albumin concentrations [75]. Studies in the general population have also shown that those with a history of smoking have a marked risk for microalbuminuria, indicating irreversible kidney damage [76]. Alongside the effect on smoking on renal function, this work indicates that the well-known risks of smoking on cardiovascular morbidities is concurrent in kidney patients and as such similar recommendations should be made in the non-dialysis CKD population as those suffering from cancer and cardiovascular disease states. Appropriate guidance is available on smoking cessation from NICE: https://cks.nice.org.uk/smoking-cessation and https://cks.nice.org.uk/smoking-cessation#!scenario:1.

The effect of alcohol misuse in a non-dialysis population is more difficult to define. The current evidence base looks to break alcohol consumption down into two categories, those that suffer from excess alcohol intake and those who consume light to moderate levels of alcohol. Recent studies suggest that in CKD populations as one patient group, approximately 20–36% of patients consume alcohol in light or moderate quantities with approximately 10% showing behaviours classified as excess alcohol intake [77]. Research suggests that alcohol intake within the recommended guidelines for the general population should not further exacerbate their condition, though decisions should be made on a per patient basis, with those with other co-morbidities (e.g. Diabetes) requiring greater considerations [77]. Access to counselling, addiction services and rehabilitation should be available. Appropriate guidance is available on smoking cessation from NICE: https://cks.nice.org.uk/alcohol-problem-drinking#!scenario.

## Haemodialysis

### Introduction

Physical activity and exercise recommendations for individuals with end-stage kidney disease receiving haemodialysis. The strengths of the recommendations and the level of supporting evidence are coded as previously using the Modified GRADE system [78] as recommended by Renal Association guidance.

### Physical activity and exercise

#### Recommendations

2.1 We recommend that physical activity and exercise should be encouraged in the haemodialysis population where there are no contraindications (1C)
2.2 We recommend that haemodialysis patients should aim for 150 min of moderate intensity activity a week (or 75 min of vigorous activity) or a mixture of both as per the UK Chief Medical Officers’ Guideline. This may include a combination of exercise outside of dialysis (interdialytic) or exercise during dialysis (intradialytic) (1B).
  We suggest that sufficient physical activity may reduce risk of cardiovascular related and all-cause mortality in the haemodialysis population (1C).
  We suggest that increased physical activity or exercise may have favourable effects on blood pressure (2C).
2.3 Exercise during haemodialysis (intradialytic exercise) is safe with no contraindications; we therefore recommend that it should be available in all units:
  To improve cardiovascular health and physical function (1B).
  To improve muscular strength (2C).
  Reduce hospitalisations (2C)
  To improve blood pressure control (2C).
  To improve lipid profiles (2D)
  To improve dialysis efficiency (2D).
2.4 We suggest that programmes for increasing physical activity and exercise are supervised and led by individuals qualified to deliver exercise and/or rehabilitation programmes in populations with chronic disease (2D).
2.5 We recommend that individual participant and staff barriers need to be addressed to optimise programme participation and adherence (1C).

### Audit measure

1. The availability of a programme for intradialytic exercise, the resource available (equipment, physiotherapist time), and the proportion of in-centre patients engaging with and maintaining regular intradialytic exercise.

### Rationale

Physical activity levels are low in haemodialysis patients [79]. Data from the Dialysis Outcomes and Practice Patterns Study (DOPPS) has reported that 43.9% (*n* = 9176) of haemodialysis patients perform no physical activity or exercise [80]. Unsurprisingly, low levels of physical activity are associated with poor health-related quality of life (HRQoL), symptoms of depression [80], and increased mortality rate in this population [80–82]. The factors that relate to these low levels of physical activity are unclear; however reductions in lean body mass [83], aging [84] and the numerous comorbidities [85] present in this population are all believed to play a role. Moreover, the haemodialysis treatment itself exacerbates these low levels, with physical activity levels reported to be lower on dialysis compared to non-dialysis days [86]. However, mortality risk has been shown to be lower in haemodialysis patients who are more physically active compared to those who are sedentary [80, 81, 87], indicating a benefit of even small modifications in physical activity in this highly sedentary population. Unfortunately, there is no RCT data on the effect of increasing physical activity levels and the association with mortality in the haemodialysis population. Although, as recommended by the most recent Kidney Disease Improving Global Outcomes (KDIGO) guidelines [88] and the UK Chief Medical Officers’ Guideline [89] increasing physical activity levels should be encouraged (aiming for at least 30 min of moderate intensity activity, 5 times per week). It is also important to highlight that even small increases in physical activity levels is likely to provide some benefit.

Data from one of the largest RCTs in the area (*n* = 296) showed that increasing physical activity levels outside of haemodialysis treatment through a 6-month personalised walking exercise programme improves physical function as measured by the 6-min walk test performance (an improvement of 39 m was reported in the exercise group) and HRQoL [90]. The participants with the highest adherence had the largest improvement in performance, and no serious adverse events or safety flags were reported [90]. This indicates that on the limited current evidence increasing levels of physical activity outside of haemodialysis is safe. In data from this trial [90], limited to participants who completed the trial, there was a significant reduction in hospitalisation in the exercise compared to the control group. The walking exercise programme was supervised by a rehabilitation team, and participant self-reported compliance was 83%. A further trial reported no significant effect on the 6-min walking test following 6-months of unsupervised home-based walking [91]. However, there was a greater increase in 6-min walk test performance in the home-based walking group (49 m) compared to usual care (where there was also a small improvement of 21 m), with the magnitude of the improvement being greater than minimal clinically important difference for this outcome [92]. Furthermore, there was analysable data for only 15 participants in both groups with the authors acknowledging that they were ultimately underpowered for their primary outcomes [91]. Finally, a non-randomised, non-controlled study of a 12 week combination of supervised class and home based exercise resulted in improvements in measured physical function (including the incremental shuttle walk test, the timed up and go test and the sit-to-stand 60) and self -reported physical activity [93]. Taken together, to date there is limited high quality RCT data on the efficacy of increasing physical activity levels outside of haemodialysis on outcomes.

Individuals receiving haemodialysis are highly sedentary (low physical activity levels) particularly on days when they receive their haemodialysis treatment. There are benefits and disadvantages to both programmes of intradialytic exercise or exercise/physical activity taking place outside of haemodialysis treatment (interdialytic). However, it is currently not clear whether one is superior to the other with regards to benefits for clinical and patient reported outcomes (including mortality cardiovascular, physical function and health-related quality of life) [94]. However, increasing and maintaining exercise behaviour in the sedentary haemodialysis population is challenging, therefore to initially encourage an increase in levels of exercise and physical activity in general, supervised intradialytic exercise (alongside other lifestyle and behaviour change advice) may be preferable (i.e. supported environment, no extra burden on time, exercising with peers). This has been highlighted by its inclusion in the latest Renal Association Clinical Practice Guideline on Haemodialysis [95]. Future trials may wish to directly compare the clinical, cost benefits and acceptability to participants of intradialytic and programmes of exercise taking place outside of haemodialysis directly.

A recent RCT in 130 participants receiving prevalent haemodialysis has indicated that a six-month programme of 30 min moderate intensity (at an RPE of 12–14) intradialytic cycling was able to reduce left ventricular mass (between group reduction of − 11.1 g, *P* < 0.001 for between group change) and improve other measures of cardiovascular health compared to a usual care control group [96]. This is in agreement with the results of two smaller studies showing the benefits of single intradialytic cycling sessions on cardiovascular health [97, 98]. The six-month programme in the aforementioned RCT [19] was delivered and supervised by trained members of the research team and reported exercise programme adherence levels to be > 70% [96]. Furthermore, a cost analysis of this trial showed that the six-month programme of intradialytic cycling was cost-effective (which appeared to be driven by a reduction in hospitalisations) [99] - this analysis included the costs associated with implementing the intervention (equipment and staff). This may be important as a recent international survey of nephrologists reported that the leading barrier to implementation of exercise programmes at haemodialysis units was funding [100]. The benefits of aerobic intradialytic cycling have been supported by a recent systematic review and meta-analysis indicating that solely intradialytic cycling results in a significant improvement of 87.84 m in six-minute walk test performance and a non-significant improvement of 1.19 mL/kg/min in V̇O_2peak_ [101]. Further systematic reviews have confirmed this by showing that aerobic based intradialytic interventions [102, 103], and exercise interventions comprised of aerobic, resistance or combinations of these exercise modalities [102–106] results in improvements in the six-minute walk test and V̇O_2peak_. Improvements in V̇O_2peak_ in this population may be of particular significance as it has been shown that values below 17.5 mL/min/kg are associated with increased mortality [107]. However, currently there is no RCT data to indicate that intradialytic exercise can reduce the risk of mortality in the haemodialysis population.

There are a number of recent systematic reviews [101, 104, 105, 108, 109] assessing the efficacy of exercise interventions (predominantly those involving aerobic exercise, resistance exercise or a combination) on HRQoL in the haemodialysis population. When assessing HRQoL some systematic review data [104, 108, 109] but not all [101], have reported improvements in the physical component score of the short form-36 following programmes of exercise. The inclusion of a range of heterogenous interventions (e.g., intra and inter dialytic, aerobic and resistance programmes (or combinations)), and methods of assessing HRQoL in the systematic review data makes providing firm guidelines for this outcome (and others) difficult. Results from the recent PEDAL trial, which investigated the effect of a 6-month programme of intradialytic exercise on Kidney Disease Quality of Life Short Form Physical Composite Score (PCS) in 335 randomised participants demonstrated that aerobic-only intra-dialytic cycling did not statistically improve HRQoL in a deconditioned population receiving haemodialysis therapy [110]. Moreover, the recent Cycle-HD trial [96] (which was not powered for HRQoL) also reported that a 6-month programme of aerobic intradialytic cycling did not statistically improve the EQ-5D-5L score, or both the physical and mental component scores of the SF-12. Resultantly, we have not provided a recommendation for HRQoL in this guideline.

Intradialytic cycling exercise delivered by means of cycle ergometer is the most prevalent modality of exercise delivered (usually performed three times a week) as part of clinical care [111] and is the most common intervention in trials of exercise in this population [112]. There may be benefit of adding an additional resistance training component to a programme of intradialytic exercise to improve muscle strength [113]. This may be important as there is a reported association between increased muscle mass and improved survival in the haemodialysis population [114, 115]. Promising results from a small, randomised pilot study have indicated that a 12-week programme of resistance training resulted in an increase in thigh muscle volume of 193 (63 to 324) cm^3^ mean difference (95% CI) [116]. Although in general the evidence for resistance training only is less clear and depending on the outcome measure it does not always provide additional benefit compared to aerobic training alone [103, 105, 117]. A consideration that must be made when adding a resistance training component is that it may require more supervision than aerobic training alone, and for this reason providing it in clinical care may be more challenging than intradialytic cycling alone.

Interventional trials have consistently demonstrated that physical activity or exercise is effective in reducing blood pressure [118]. The relationship between blood pressure and outcome in dialysis patients is “U”-shaped [119], that is high blood pressure associates with mortality, whilst low blood pressure is even more strongly associated with adverse outcomes [120]. Therefore, effects of exercise on blood pressure in the haemodialysis population should be interpreted with this in mind. Systematic review data on the effect of exercise on blood pressure in the haemodialysis population is mixed. Some systematic reviews and meta-analyses have shown that intradialytic exercise training may reduce blood pressure [103, 104, 109], whilst others have reported either no effect [105, 121] of exercise training or a very small non-significant reduction [121]. A recent randomised controlled trial of 130 participants reported a non-significant reduction of 4.9 mmHg in interdialytic systolic blood pressure in the exercise group (there was also a reduction in the control group) following a 6-month programme of intradialytic exercise [96]. However, there was no change in blood pressure following a 6-month personalised home-based walking programme in 104 participants randomised to the exercise group in a previous RCT [90]. This supports an earlier interventional trial showing no effect of either intradialytic or home-based aerobic exercise interventions on blood pressure [91]. The current evidence base for the exercise or physical activity inducing favourable changes in blood pressure in the haemodialysis population is weak.

The limited systematic review and meta-analysis data to date show that exercise training had no effect on circulating total cholesterol [103, 104], supported by RCT data showing no effect of a 6-month home based walking programme on circulating cholesterol or triglyceride [90]. To date there is no strong data that exercise or physical activity interventions may lower circulating lipids. Moreover, it is not clear whether small changes in lipid profiles would result in meaningful changes in outcomes given the role of lipids in the pathogenesis of cardiovascular disease in this population. It has been suggested that exercise training during dialysis (intradialytic exercise) may improve dialysis efficiency (Kt/V_urea_) through increases in skeletal muscle blood flow which may reduce the rebound of solutes [122]. Although this has yet to be consistently shown in RCTs. Some systematic reviews and meta-analyses have shown an improvement in Kt/V_urea_ with intradialytic exercise [103, 104] whilst a recent systematic review found no effect in seven out of 13 included studies, which suggested little to no effect on dialysis clearance [123]. There is limited data on the effect of exercise on medication. An observational study has shown that a 6-month intradialytic exercise programme resulted in a reduction in antihypertensive medication and weekly dose of erythropoietin [124]. Furthermore, a recent cost-effective analysis of an RCT showed a reduction in mean cost of medication after a 6-month intradialytic exercise programme [99]. Currently, there is limited evidence to provide recommendations for the effect of exercise on medication.

#### Evidence for the safety of exercise

Systematic review data has reported no significant serious adverse events due to exercise training, citing this as evidence of safety [109, 125, 126]. However, a previous systematic review [101] has highlighted inconsistencies in adverse event reporting in trials of exercise in the haemodialysis population. There have been safety concerns that exercise during dialysis may exacerbate the detrimental effect of the haemodialysis process. However, a recent RCT has reported that intradialytic cycling did not increase the number of arrhythmias during and following haemodialysis treatment [96]. In addition, the six-month programme of intradialytic cycling which was employed in this RCT was associated with favourable cardiovascular remodelling [96], which also suggests no detrimental effects. A primary concern for performing intradialytic exercise is the precipitation of intradialytic hypotension; this is of concern as episodes of intradialytic hypotension are associated with poor outcomes and increased mortality [127], with intradialytic hypotension being present in around 10% of total sessions [128]. Data from a small, randomised controlled crossover trial of 15 participants [129] demonstrated that despite blood pressure increases during intradialytic cycling there is a resultant period of asymptomatic hypotension in the period following exercise. Reassuringly, this was not associated with changes in humoral markers of cardiac disease or systemic inflammation (including hsTroponin I, IL-6 or TNF-α) [129]. The reduction in blood pressure observed following exercise in this trial [129] likely reflects a normal physiological response to exercise. Traditionally, it has been believed that exercise should be avoided in the second half (the last 2 h) of the haemodialysis treatment, particularly in individuals who are having a large amount of fluid removed [130]. However, in a recent multi-centre randomised crossover trial which included 84 participants, there was no significant difference between rate of intradialytic hypotension per 100 haemodialysis hours when exercise was performed in the first half compared to the last half of treatment [131]. This supports data from another smaller mechanistic crossover study [132], which showed that intradialytic cycling did not exacerbate instability during haemodialysis treatment when conducted in the first or third hour of treatment, independent of participant hydration status [132]. The current evidence base indicates that intradialytic exercise is safe and is not associated with increased cardiovascular risk. A large RCT involving intradialytic exercise and hard outcomes (i.e., mortality) may be needed to provide conclusive answers regarding the safety of intradialytic exercise.

In summary, it is recommended that intradialytic exercise be performed three times a week, for at least 30 min. It is important to note that performance of intradialytic exercise three times per week (as performed in all the intradialytic exercise trials) is still not sufficient to meet the recommended levels (a 150 min of moderate intensity activity a week (or 75 min of vigorous activity)) of physical activity in recommendation 2 of this guideline. Therefore, intradialytic exercise will require supplementing with exercise or physical activity activities performed outside of the haemodialysis setting (interdialytic) to meet recommended UK Government guidelines.

### Implementing intradialytic exercise

We suggest the following guidance for implementing intradialytic exercise at haemodialysis units. These are modified from the guidelines for implementing intradialytic exercise provided in the Renal Association Clinical Practice Guideline on Haemodialysis [95].

- Exercise should be supervised for greatest compliance and efficacy by an appropriately trained individual (e.g., physiotherapist, exercise scientists, cardiac rehabilitation specialist or an assistant physiotherapist/dietitian/nurse with additional training from one of the former groups).
- Exercise should be provided in the form of intradialytic cycling, delivered by a static cycle ergometer.
- Exercise should be completed for at least 30 min during every haemodialysis treatment (three times per week). We suggest avoiding the first 30 min of treatment.
- Exercise should be performed at a moderate exercise intensity. This should be between 12 and 14 on the Borg RPE Scale. This will enhance adoption and adherence in novel exercisers
- Exercise can be progressed gradually by increasing duration, frequency (if not exercising during every haemodialysis treatment) and intensity (through increasing the resistance on the cycle ergometer).
- There are no contraindications to performing exercise in the last half of the haemodialysis treatment
- Resistance training (e.g. TheraBands and/or lifting of ankle weights) can be added including components of lower or upper body. There is no evidence that performing light upper body resistance exercise has adverse effects on vascular access.
- Once patients are familiar with exercising during dialysis they should be encouraged to complete additional exercise on non-dialysis days.
- To maintain exercise behaviour, behavioural strategies such a social support, goal setting of outcomes, instruction (modelling) of exercise behaviours, and motivational interviewing should be implemented.
- Where possible exercise programmes should be individualised to participant needs.
- For guidance around training and development of the multi-professional weight management team, see upcoming Global Renal Exercise (GREX) training and development programme. (https://grexercise.kch.illinois.edu).
- Patients should avoid exercise:
  - ◦ Less than 3 months after initiation of haemodialysis.
  - ◦ If they have any uncontrolled medical condition (clinically unstable) including (but not limited to) infection or fever, recent (within 2 weeks) myocardial infarction or undiagnosed chest pain.
  - ◦ If they have any perceived physical or psychological barriers to exercise participation.
  - ◦ In patient in class D (unstable condition) as per the American Heart Association/American College of Sports Medicine Joint Position Statement: 1) unstable ischemia; 2) heart failure that is not compenstated;3) uncontrolled arrhythmias; 4) severe and symptomatic aortic stenosis; 5) hypertrophic cardiomyopathy or cardiomyopathy from recent myocarditis; 6) severe pulmonary hypertension; or 7) other conditions that could be aggravated by exercise (for example, resting systolic blood pressure > 200 mmHg or resting diastolic blood pressure > 110 mmHg; active or suspected myocarditis or pericarditis; suspected or known dissecting aneurysm; thrombophlebitis and recent systemic or pulmonary embolus).
  - ◦ Symptomatic hyper- or hypotension.
- We suggest the following safety monitoring:
  - ◦ Prior to exercise, ask the patients how they feel, record last measured intradialytic blood pressure and heart rate.
  - ◦ During exercise, ask patient to report symptoms of pain, excessive fatigue, altered consciousness, overheating, anxiety, severe breathlessness, chest pain, dizziness / light-headedness.
  - ◦ Rating of perceived exertion scale can be used during exercise to monitor intensity and ensure exercise intensity does not provoke response greater than 15/hard (heavy) on the Borg RPE scale.

### Weight management

#### Recommendations

2.6 We recommend that regular anthropometric measurements should be taken to assess changes in body composition [1B]
2.7 We recommend that all individuals receiving haemodialysis maintain a BMI of between 20 and 30 kg/m2 (1C).
2.8 We recommend that a multi professional approach should be taken to weight management. This should include the evaluation of nutritional needs along with comorbid conditions, and the promotion of physical activity and exercise supported by behaviour change techniques (2C).
2.9 We suggest that bariatric surgery is safe and may be considered for those individuals wishing to receive a transplant for whom current BMI prevents this (2C).

#### Audit measure

1. Regular monthly assessment of accurate body mass and BMI via appropriate methods (clinical judgement can be used to identify the appropriate method).

#### Rationale

Data from observational studies indicate that obesity is an independent risk factor for chronic kidney disease [133, 134]. Moreover, in the general population and in kidney transplant recipient’s obesity is an established risk factor for cardiovascular disease and mortality [64, 135]. However, in the end-stage kidney disease (ESKD) population who are receiving haemodialysis as a form of renal replacement therapy, several large observational studies [136–138] have shown that the relationship between obesity and mortality is paradoxically in the opposite direction. That is, that higher BMI is associated with increased survival (termed the obesity paradox) [115]. One of the first studies to describe this relationship using United States Renal Data System (USRDS) data showed that haemodialysis patients with low BMI have a significant increased chance of mortality, whereas incremental increases are associated with improved mortality risk over a 5 year follow up [137]. A further study characterised levels of BMI in 1356 haemodialysis patients into underweight (BMI < 20 kg/m^2^), normal weight (BMI 20 to 27.5 kg/m^2^), and overweight (BMI > 27.5 kg/m^2^) [138]. They demonstrated that compared with normal weight (BMI between 20 to 27.5 kg/m^2^) being underweight (< 20 kg/m^2^) was associated with a significantly higher chance of mortality, whilst being overweight (> 27.5 kg/m^2^) improved survival [138].

This relationship was further observed in a study of 418,055 haemodialysis participants who initiated treatment between 1995 to 2000 [139]. They reported a significant improvement in overall 2-year survival by increasing BMI [139]. After adjustment for cardiovascular risk factors a BMI of 25 kg/m^2^ or greater was associated with decreased mortality compared with a BMI of 22–25 Kg/m^2^. Conversely a BMI of less than 22 kg/m^2^ was associated with the greatest risk of mortality [139]. A further study of data from the Dialysis Outcomes and Practice Patterns Study (DOPPS) which included 9714 haemodialysis patients from the USA and Europe found that increasing BMI is associated with improved survival in the haemodialysis population [140]. In agreement with previous observations [138], a BMI of below 20 kg/m^2^ was associated with a higher risk of mortality [140]. Interestingly, the lowest mortality risk was reported in the BMI ≥30 kg/m^2^ category [140]. A further study of data from 54,535 haemodialysis patients in the US also reported that increasing BMI is associated with both a reduced all-cause and cardiovascular mortality in haemodialysis patients following a 2-year follow up period [141].

It is important to note that the association between increased BMI and reductions in mortality have not been observed in all investigations. For example, De Mutsert et al. [142] followed 722 Dutch haemodialysis patients over 7 years and found that a baseline BMI of 30 kg/m^2^ was associated with an increased risk of mortality compared to a BMI of 22.5 to 25 kg/m^2^. Moreover, the relationship between BMI and mortality may be dependent on other factors such as age [143]. This was supported by a study which reported that obesity was a stronger risk factor for mortality in 984 younger (< 65 years) peritoneal and haemodialysis patients, compared to 765 patients over 65 [143]. They reported that patients younger than 65 at the start of dialysis with a BMI above 30 kg/m^2^ had a 70% higher risk of mortality compared with a normal BMI (20–24 kg/m^2^). Reporting a U-shaped association between BMI and mortality [143], i.e. a greater risk in the extreme low and high BMI categories as per data from the general population.

Whilst recognising the limitations of observational data (there is no RCT data available), there is reported association between a higher BMI and improved survival in the haemodialysis population. A major limitation of the majority of these studies is there short-term (≤5 years) follow up [137, 139]. The < 5-year survival of individuals receiving haemodialysis is small [144], and consequently the long-term survival associated with lower levels of obesity may (may be overwhelmed by the short-term effects of PEW and inflammation in this population). Studies in the general population that show increased mortality in overweight and obese adults compared with normal BMI have follow up durations in excess of 5 years [145, 146]. Two previous studies [142, 147] in the haemodialysis population who have followed up individuals receiving haemodialysis for 7 and 12 years reported that increased BMI was associated with increased mortality.

Further explanation for the obesity paradox may be explained by the limitations of BMI in differentiating between muscle and fat mass [148]. Increased fat mass (particularly visceral fat) is associated with inflammation in the ESKD population [149], inflammation is strongly related to mortality in the dialysis population therefore adiposity is unlikely to confer a survival advantage. The negative consequences of visceral fat are supported by data that showed that abdominal obesity and waist circumference were stronger predictors of all-cause mortality and cardiovascular death than BMI in 537 individuals receiving haemodialysis [150]. In this study, higher BMI was protective, however in contrast a higher waist circumference was a predictor of higher mortality [150]. The importance of increased muscle mass in the haemodialysis population has been shown by a series of studies [114, 115, 150–152]. In a study that controlled for muscle mass (by using measures of creatinine production), it was found that regardless of BMI, those with high muscle mass had higher survival rates than those with low muscle mass [114]. Suggesting, that increased muscle mass in haemodialysis patients is protective against mortality [114]. Another study [115] of 50,381 individuals receiving haemodialysis who survived 6 months found that weight loss with an increased serum creatinine (indicating maintenance of muscle mass) was associated with greater survival than weight gain but a decrease in creatinine levels (suggesting weight gain but muscle mass loss). These observations were confirmed in a study [151] that showed that higher mid-arm muscle circumference (a measure of muscle mass) was associated with a trend towards increased survival in 1709 individuals with ESKD receiving haemodialysis. Further associations with increased fat mass and lean mass (which was defined as BMI minus fat mass index) and increased survival has been reported in 808 Japanese haemodialysis patients following a 53 month follow up period [152]. Moreover, in a large cohort of 117,683 haemodialysis patients estimated lean body mass (through creatinine-based equations) was linearly associated with lower mortality [153]. Taken together, the available data appear to show that high muscle mass is protective against mortality, whilst loss of muscle mass (particularly with an increase in fat mass) has negative consequences for individuals receiving haemodialysis. Therefore, individuals receiving haemodialysis should avoid muscle mass loss.

Lastly, although some studies have shown that a BMI of above 30 kg/m^2^ is associated with a survival benefit, many haemodialysis patients will be precluded from obtaining a kidney transplant if they are in this BMI category. Indeed, this is supported by data showing greater rates of surgical wound infections, delayed graft function and acute rejection in obese kidney transplant recipients compared to those who are not obese [154–156]. Graft survival time has been shown to exceed 80 months in around 50% of morbidly obese transplant recipients compared to 70% of recipients with an ideal BMI [154]. Moreover, a pooled analysis has shown that higher pre-transplantation BMI is associated with a higher mortality in kidney transplantation recipients [157]. Adjusted annual rates of survival in kidney transplant recipients are up to 200% greater than individuals who remain on the kidney transplant waiting list [154, 158]. These rates of survival following transplantation are greater than those conferred by higher BMI in the haemodialysis population, showing that the association between high BMI and improved survival does not equal the marked increase in survival that transplantation confers. Therefore, the suitability for transplantation should be the optimal consideration when assessing weight in individuals receiving haemodialysis for whom transplantation is attainable.

For those individuals for whom transplantation is not attainable based on BMI, there is some evidence that weight loss through bariatric surgery may improve kidney transplant access, and in turn long term outcomes, in particular if non-surgical measures have proved unsuccessful. This is highlighted by a recent KDIGO guidelines which suggests that bariatric surgery should be considered as an option to achieve a BMI < 30 kg/m^2^, and therefore suitability for transplant [159]. Previously, there has been concerns relating to aggressive weight loss (based around the obesity paradox), in addition to a higher surgical risk (particularly for those with advanced CKD [160], which traditionally has reduced the referring of individuals with ESKD for bariatric surgery. Despite this, a recent study which analysed Medicare claims data in the US to identify bariatric surgery in individuals with ESKD found that between 2006 to 2016 there was a nine-fold increase in the overall number of bariatric surgeries [161], with laparoscopic sleeve gastrectomy being the most prevalent type of bariatric surgery [161]. Although a previous report has observed higher 30-day risk of reoperation, readmission and mortality compared to patients without CKD [162], Sheetz et al. [161] reported no difference in 30-day postoperative complications between patients with and without ESKD. The patients with ESKD did have a slightly longer length of stay, and readmissions were higher (8.6%) for ESKD patients compared to those individuals without (5.4%) [161]. In a follow up study observing USRDS data from 2006 to 2015 (and comparing to nonsurgical control patients) bariatric surgery was associated with lower all-cause and cardiovascular mortality at 5 years [163], which is at odds with the obesity paradox. Interestingly, bariatric surgery was associated with an increase in kidney transplantation at 5 years [163]. This is in agreement with data from another prospective study showing that over half of obese participants achieve a target BMI suitable for transplant following laparoscopic sleeve gastrectomy [164]. It is important to note that a clinical consideration that must be made prior to bariatric surgery is the need for the individuals to demonstrate commitment to a sustained period of lifestyle modification. This is in line with the NICE recommendation that candidates for bariatric surgery should have previously undergone a service based weight loss programme for at least 6 months (https://www.nice.org.uk/guidance/cg189/chapter/1-Recommendations#physical-activity).

Taken together, these data from cohort studies tentatively suggest that bariatric surgery may have long-term health benefits for individuals with ESKD, and its use for weight loss to facilitate access to transplant may be considered, although added risk of this procedure in the haemodialysis population cannot be currently discounted based on the available evidence. Future definitive RCT data is needed to ascertain the balance between the effectiveness of this treatment (for improved outcomes including accessibility for transplant) and any potential long-term harm.

### Implementing weight loss in the haemodialysis population

We suggest the following guidance for encouraging and monitoring weight loss in haemodialysis patients:

- In individuals receiving haemodialysis it is reasonable for a registered dietitian to use clinical judgement to determine the most effective way to measure body weight or composition. For guidance, please see the recent KDOQI clinical practice guideline for nutrition in CKD: 2020 update [165].
- The standard weight status categories that have been defined by the World Health Organisation (WHO) according to BMI ranges for adults can be used in the haemodialysis population; these include < 18.5 kg/m^2^ for underweight; 18.5 to 24.9 kg/m^2^ for normal weight; 25.0 to 29.9 kg/m^2^ for overweight; and ≥ 30 kg/m^2^ for obese.
- BMI as maker for weight loss in the haemodialysis population is limited as it cannot differentiate between fat and muscle mass. Measures of body composition may be more informative
- Weight loss should be discussed with persons who would be eligible for transplant except for their degree of obesity.
- Weight loss programmes should be individualised wherever possible and take into account body composition, the aim of interventions should be to increase muscle mass in conjunction with reducing fat mass. Muscle mass loss should be avoided in programmes of weight loss.
- Programmes of weight loss require a multidisciplinary approach (which should include other healthcare providers such as dietitians, physiotherapists and health psychologists), and should evaluate nutritional needs along with comorbid conditions. This should be in conjunction with the promotion of physical activity and/or exercise.

### Other lifestyle considerations (smoking, alcohol intake, drug use)

#### Recommendations

2.10 We recommend that individuals receiving haemodialysis stop smoking (1A).
2.11 We recommend alcohol consumption should be within national guidelines (1B).
2.12 We recommend that individuals receiving haemodialysis avoid all recreational drug use (1B).

#### Audit measures

1. Proportion of individuals receiving haemodialysis who smoke.
2. Proportion of individuals referred (including self-referral) to a smoking cessation programme.
3. Number of individuals receiving haemodialysis with excess alcohol intake and referred on to support services.

#### Rationale

Individuals with established cardiovascular disease at haemodialysis inception are more likely to be former smokers. A large database study in individuals receiving haemodialysis has shown that current smoking is associated with cardiovascular disease and mortality [166]. This was indicated by a higher incidence of heart failure, peripheral vascular disease, and mortality in smokers compared to non-smokers [166]. These findings demonstrating a higher risk of cardiovascular disease and mortality in the haemodialysis population have been confirmed in a subsequent database study and systematic review [167, 168]. This mirrors the well-known association in the general population between smoking, cancer and cardiovascular disease.

A previous cross-sectional study in 163 individuals with ESKD receiving haemodialysis found that excess alcohol intake was present in 45 (27%) of participants and may be associated with poor nutrition, hypertension and concomitant liver disease [169]. Similar levels of alcohol and drug dependency have been reported in another study from a Veterans Affair dialysis unit [170]. Further studies are required to confirm these findings and ascertain the effect of increased alcohol intake on patient outcomes in this population. Alcohol use within recommended guidelines for participants receiving haemodialysis is likely to be safe. Recreational drug use can increase the risk of ESKD [170], and can result in hypertension [170, 171], moreover some recreational drugs such as cocaine are associated with an elevated risk of cardiovascular complications [172]. For these reasons recreational drug use should be avoided in the haemodialysis population.

## Implementing smoking cessation and alcohol intake guidance in the haemodialysis population

We suggest the following guidance for encouraging smoking cessation in the haemodialysis population:

- Appropriate guidance on smoking cessation is available from NICE https://cks.nice.org.uk/smoking-cessation and https://cks.nice.org.uk/smoking-cessation#!scenario:1
- Appropriate guidance on alcohol misuse can be found at https://cks.nice.org.uk/alcohol-problem-drinking#!scenario

## Transplantation

### Introduction

Kidney transplantation is the preferred form of renal replacement therapy (RRT) for patients with end-stage renal disease (ESRD) [173]. Kidney transplantation often results in beneficial effects on quality of life (QoL) [174] and overall survival rate [175] when compared to dialysis. Nonetheless, kidney transplanted patients are burdened by high cardiovascular risk due to the increased prevalence of traditional but also disease-specific cardiovascular risk factors [6, 173]. Weight gain, obesity, diabetes, hypertension, and metabolic syndrome are predominant features in these kidney transplanted patients and are associated with worse outcomes, including premature death, cardiac events and graft loss [176–179]. Cardiovascular disease (CVD) remains one of the leading causes of death in kidney transplant recipients (KTRs), accounting for 17% [180] of total deaths. KTRs have an overall mortality rate of approximately 5–10-fold greater than the general population [181]. Also, immunosuppressive therapy may contribute to the development of dysmetabolism and worsening of sarcopenia [182], and low muscle mass has been associated with poor survival after kidney transplantation [183].

Appropriate self-management and a healthy lifestyle are recommended to KTRs and represent relevant aspects of the clinical care aiming to control these key cardiovascular risk factors and to preserve the long-term graft function. A core component of generalised lifestyle advice is the promotion of physical activity. Physical inactivity is one of the major risk factors for mortality in the general population [6] and multiple studies have shown that in the general population, physical activity is associated with a less deleterious CVD risk-factor profile and consequently fewer adverse cardiovascular outcomes [7, 184]. Increasing exercise and physical activity levels is an attractive option for addressing many of the underlying CVD risk factors in KTRs.

We first reviewed and summarised current epidemiological evidence that has investigated either physical activity or exercise levels in KTRs and/or the association physical activity and exercise levels with outcomes. A systematic search of existing systematic and narrative reviews of physical activity and exercise in KTRs was conducted. NCBI MEDLINE (1966-present day) was searched using the following MESH search terms: kidney transplantation; transplant recipients; exercise; exercise therapy. An example of a full search strategy can be found in Additional file 1: Appendix 1. To gather the most recent evidence available, only reviews published in the last 5 years were sought (2015 to 2020). After full-text review, a total of 14 reviews relating to physical and activity in renal transplant recipients were identified. These reviews were hand-searched and we sought each review for appropriate information, references of studies, and data pertaining to physical activity and exercise levels in KTRs, and the association with outcomes. Secondly, we conducted, where appropriate, a pragmatic hand-search of all current guidelines and position statements pertinent to lifestyle, physical activity, and exercise levels in KTRs.

Lastly, whilst there have been previous systematic reviews investigating the effect of exercise and/or physical activity interventions in KTRs, many are now outdated [185–187] and two previous meta-analyses have been completed on the subject area [188, 189]. As such, a new systematic search and meta-analysis of randomised clinical trials studying the effect of receiving a physical activity or exercise intervention, either supervised or unsupervised, on outcomes in patients with (or awaiting) a kidney transplant. The following electronic databases were searched from their date of establishment to January 2020: National Centre for Biotechnology Information (NCBI) PubMed (which includes the Medical Literature Analysis and Retrieval System Online (MEDLINE)), and the Cochrane Central Register of Controlled Trials (CENTRAL) (includes Excerpta Medica database (EMBASE), and the WHO International Clinical Trials Registry Platform (ICTRP)). The following MESH search terms were used to search all databases: kidney transplantation; transplant recipients; exercise; exercise therapy; randomised controlled trial. Full search strategies can be found in Additional file 1: Appendix 2. A flow of information through the different phases of the search can be found in the figure in Additional file 1: Appendix 3. Complete tables (Additional file 1: Appendix 4), forest plots (Additional file 1: Appendix 5), risk of bias summary (Additional file 1: Appendix 6), ‘leave-one-out’ sensitivity analysis (Additional file 1: Appendix 7), and funnel plots (Additional file 1: Appendix 8) relevant to this meta-analysis can be found in the appendices.

The strengths of the recommendations and the level of supporting evidence are coded as previously using the Modified GRADE system [78].

### Physical activity and exercise

#### Recommendations

3.1 We recommend that general physical activity should be encouraged in KTRs without contraindications [1B]
3.2 We suggest sufficient physical activity, pre- and post-transplant, can reduce all-cause and cardiovascular mortality [2C]
3.3 We recommend KTRs aim for 150 min of moderate to vigorous physical activity a week (or 75 min vigorous physical activity) as per the UK Chief Medical Officers’ Guideline [1C]
3.4 We suggest individual barriers and activators to physical activity need to be identified and addressed to optimise programme uptake and adherence [2C]
3.5 We recommend that structured exercise be considered as a method of enhancing cardiorespiratory fitness [1B]
3.6 We recommend that structured exercise be considered as a method of enhancing muscular strength and physical function [1C]
3.7 We suggest that structured exercise be considered as a method of improving health-related quality of life and increasing HDL levels [2C]
3.8 Structured exercise alone is not sufficient to attenuate increases in body mass following transplantation; we therefore suggest a multi-professional approach to appropriate weight-management strategies [2B]
3.9 We suggest that structured exercise should be performed at least 3x/week in KTRs without contraindications [1C]
3.10 We suggest that KTRs without contraindications undertake both aerobic and resistance exercise to maximise the effects on exercise capacity and muscle function [1B]
3.11 We suggest that a structured exercise routine be devised (and supervised if possible) by appropriately trained staff [2B]
3.12 We suggest exercise programmes should be individualised based on underlying patient goals/expectations, pathophysiology, level of experience, and graft status [2C]

#### Audit measures

1. Healthcare professionals should take the opportunity, whenever possible, to identify inactive patients and levels of physical activity should be routinely checked. This could be by simply asking the patient about their activity levels or via a formal validated screening tool such as the Physical Activity Vital Sign (PAVS) (endorsed by the American College of Sports Medicine (www.acsm.org) Exercise is Medicine®). The PAVS consist of two questions:
  *“On average, how many days per week do you engage in moderate to strenuous exercise like a brisk walk?”*
  *“On average, how many minutes do you engage in exercise at this level?”*

The PAVS is highly associated with decreased levels of BMI and odds of obesity and has been tested for face and discriminant validity [190].

2. Alternatively, physical activity status may be assessed by the General Practice Physical Activity Questionnaire (GPPAQ) – a NICE recommended survey to help identify those inactive and need of support. All patients who receive a score of less than ‘active’ should be provided with appropriate advice to increase their physical activity levels or offered a Brief Intervention in Physical Activity in line with the NICE Guidance (2006) (https://assets.publishing.service.gov.uk/government/uploads/system/uploads/attachment_data/file/192453/GPPAQ_-_guidance.pdf).
3. Healthcare professionals should help patients identify their circumstances, preferences and barriers to being physically active. The NICE ‘Physical activity: brief advice for adults in primary care’ (PH44) has recommendations on how to deliver and follow up up on brief physical activity advice: https://www.nice.org.uk/guidance/ph44/chapter/1-Recommendations#recommendation-2-delivering-and-following-up-on-brief-advice

#### Implementing physical activity and exercise guidance

We suggest the following guidance for implementation of physical activity and exercise in KTRs:

- KTRs should be encouraged to follow current UK general physical activity guidelines (150 min (2.5 h) of moderate (such as brisk walking or cycling) to vigorous (such as running) physical activity a week (or 75 min vigorous physical activity) relevant for their age (https://www.nhs.uk/live-well/exercise/ and https://www.gov.uk/government/collections/physical-activity-guidelines).
- KTRs should aim to minimise the amount of time spent being sedentary, and when physically possible should break up long periods of inactivity with at least light physical activity.
- Physical activity can comprise of general leisure-time physical activities, structured exercise, or sport, if appropriate.
- Exercise should be supervised for greatest compliance and efficacy by an appropriately trained individual (e.g., physiotherapist (including specialist renal if available), sport scientist, cardiac rehabilitation specialist or an assistant physiotherapist/dietitian/nurse with additional training from one of the former groups).
- Aerobic exercise should be performed at an intensity of > 60% of maximum (either based on heart rate or VO_2_peak) in KTRs without contraindications [1C]
- Resistance training, comprising of upper and lower body components, should be performed at an intensity of > 60% 1-RM at least 2x/week in KTRs without contraindications [1C]
- Exercise volume, both aerobic and resistance in nature, should be progressed gradually by adjusting duration, frequency, and/or intensity until the desired exercise goal (maintenance) is attained.
- Exercise should be followed by cool down activities (e.g. exercising for a minimum of 5 min, starting at one half of prescribed training intensity and gradually decreasing intensity until exercise is stopped).
- To maintain exercise behaviour, behavioural strategies such as social support, goal setting of outcomes, instruction (modelling) of exercise behaviours and motivational interviewing should be implemented.
- Provide information about local opportunities to be physically active for patients with a range of abilities, preferences and needs.
- For guidance around training and development of the multi-professional team, see upcoming Global Renal Exercise (GREX) training and development programme. (https://grexercise.kch.illinois.edu).

#### Rationale

Physical activity is defined as any bodily movement produced by skeletal muscles that requires energy expenditure. The term “physical activity” should not be mistaken with “exercise”. Exercise is a subcategory of physical activity that is planned, structured, repetitive, and purposeful in the sense that the improvement or maintenance of one or more components of physical fitness is the objective. Physical activity includes exercise as well as other activities which involve bodily movement and are done as part of playing, working, active transportation, household chores and recreational activities.

##### Physical activity

To measure physical activity in KTRs, recipients have been compared to the general population, other patients with CKD, and patients with other chronic diseases. The prevalence of physical activity differs widely between studies, most likely due to differences in assessment methods. As such, data on physical activity in KTRs are limited and mainly obtained by non-objective methods [6]. Overall, starting immediately pre-transplantation, levels of physical activity are generally lower in KTRs than in the general population. During the first year after renal transplantation, a partial recovery of physical activity occurs and activity is higher overall in recipients compared with patients remaining on dialysis therapy [191, 192]; however, levels remain considerably below that of age-matched healthy controls [173, 193]. Sufficient physical activity estimates range from 11 to 52% [193–197]. Wilkinson et al. [191] recently found only 27% of 2240 KTRs were sufficiently active. Many of these studies, however, are particularly prone to selection bias, as patients with poor health or with extensive comorbidities are less likely than healthier individuals to participate in such studies, potentially leading to overestimates of the level of physical activity among the patient population [193].

Small epidemiological evidence suggests that higher levels of physical activity are associated with reduced mortality [198, 199]. Rosas et al. [199] found that physical activity levels pre-transplantation predicted all-cause mortality in 507 KTRs. Here, the mortality rate for active patients was 16% compared to 36% in those deemed inactive. Post-transplantation, several observational studies support a relationship between physical activity and cardiovascular risk factors such as obesity, metabolic syndrome, dyslipidaemia, and glucose intolerance. The largest investigation to date was a large prospective study by Zelle et al. [198] who found cardiovascular mortality was inversely associated with physical activity levels, 11.7% in the lowest physical activity tertile (0–27 MET-min/d) and 1.7% in the highest physical activity tertile (234–514 MET-min/d). Higher physical activity in patients with kidney transplant may also favourably affect graft functioning, evidence which is partially supported by RCT data. Gordon et al. [194] followed the eGFR of 88 KTRs (2 months after transplant) 6 and 12 months after transplantation. Physical activity was found to be significantly associated with kidney function, with eGFR ~ 8 mL/min/1.73 m^2^ higher in physically active KTRs compared with those who were sedentary.

Many of the barriers to physical activity are similar to those observed in the dialysis population, frequently pre-dating transplantation. Such barriers include fatigue, illness, post-operative effects, medications [173], and lack of clinician guidance [173, 200]. Other barriers such physical limitation [6, 201, 202], medical comorbid conditions [194, 201, 202], fear of hurting graft incision [194], skeletal muscle atrophy [203], depression [180, 203], fatigue [201], low physical self-efficacy [202], and lack of motivation [194] may also contribute. The person prescribing physical activity/exercise to KTRs should be aware of any individual barriers and motivators.

Recent guidelines for KTRs have attempted to provide some context to lifestyle, exercise and physical activity recommendations yet are still unable to provide detailed guidance. The 2009 ‘KDIGO Clinical Practice Guideline for the Care of Kidney Transplant Recipients’ was the first published guideline to refer to a recommendation of exercise therapy [204, 205]. This guideline continues to be widely cited as support for subsequent exercise guidance in this population, yet, whilst raising awareness, does not describe exercise therapy recommendations in any detail and is therefore of limited practical application:

*“We recommend that patients are strongly encouraged to follow a healthy lifestyle, with exercise, proper diet, and weight reduction as needed”*

The evidence supporting this recommendation was graded as low quality, based on one small RCT [206]. Subsequently, the National Kidney Foundation’s KDOQI ‘Managing Transplant Recipients’ clinical guide [204] provided a commentary supporting the 2009 KDIGO Guideline, but with no additional detail. British and Australian Expert and Position Statements on exercise in CKD also endorse the KDIGO recommendations but do not provide specific guidance for transplant recipients [207, 208]. Likewise, the current National Institute for Health and Care Excellence (NICE) guideline for managing kidney disease in adults encourages self-management, including providing information about exercise, but does not provide any specific guidance beyond this, nor does it distinguish between KTRs and other stages of CKD [209]. Similarly, the recent Japanese Society of Renal Rehabilitation Clinical Practice Guideline proposes the implementation of physical activity (and exercise) therapy for KTRs [210], yet stops short of addressing the type, intensity or period. The 2017 Renal Association clinical practice guideline in post-operative care in kidney transplant recipients [211], endorsed by the British Transplant Society, suggests KTRs:

“*… participate in physical activity at a level similar to that recommended to age and co-morbidity matched counterparts from the general population as part of their lifestyle recommendations”*.

Overall, there is no evidence to suggest that KTRs should not be encouraged to follow current UK general physical activity guidelines (150 min of moderate to vigorous physical activity a week (or 75 min vigorous physical activity) relevant for their age (https://www.nhs.uk/live-well/exercise/ and https://www.gov.uk/government/collections/physical-activity-guidelines).

##### Structured exercise

With regard to structured exercise, evidence from RCTs (including the new meta-analysis conducted in the development of these guidelines) suggests that appropriate exercise interventions can improve cardiorespiratory fitness and exercise capacity [212–217]. Exercise may also increase muscular strength and physical function, although the inclusion of resistance training is important to maximise the benefits on these factors. Changes in muscle strength are likely due to improvements in muscle mass and/or metabolic functioning [214, 218], although further data are needed to support this. Exercise also can improve patient-reported outcomes including self-reported functional ability and quality of life. Exercise, of sufficient stimulus, is widely recognized to raise HDL levels [219] and our findings support that as seen in non-dialysis CKD patients. With low HDL levels associated with graft failure in KTRs, exercise may be an attractive means to increase HDL and confer positive effects on graft function [220].

Exercise appears to have beneficial effects on endothelial function, especially arterial stiffness. Arterial stiffness is an important marker of cardiovascular health and is predictive of outcome in haemodialysis patients and patients with CKD [221]. Our analysis of studies showed that exercise training resulted in a moderate (0.13 mg/dl), although a non-significant reduction, in creatinine following exercise. Differences in transplant vintage and the natural expected ‘recovery’ of renal function after transplantation may have confounded any effects of exercise.

Obesity, and weight gain, is frequently observed in patients with kidney disease post-transplantation [157, 222]. Exercise alone does not appear to alter body mass or BMI, even in a study targeted at obese patients [218]. Whilst exercise may attenuate increases in body and/or fat mass in some cases [213], the transplantation process may confound any beneficial effects of short-term exercise. Complex interventions encompassing physical activity, dietary behaviour change, and medication management warrant further investigation. This could involve a multi-professional team input of dieticians, pharmacy, and physiotherapists. Overall the effect of exercise on outcomes is confounded by typical changes post-transplant and most studies are of a small sample and short duration with a high risk of bias. Additional long-term large sample RCTs are needed to fully understand the effects of exercise in KTRs.

Based on current evidence there is insufficient evidence for the role of structured exercise to improve blood pressure, haemoglobin levels, other markers of dyslipidaemia such as glucose and triglycerides, inflammatory markers such as C-reactive protein, TNF-α, TNFR-1, TNFR-2, fetuin-A, or IL-6 values, or sleep in KTRs.

Because of the heterogeneity in interventional approaches, it is difficult to recommend or conclude which exercise modality is best. However, with most of the efficacious studies prescribing exercise at least 3x/week for a duration of 3–6 months, it is realistic to propose an exercise intervention of at least this length may provide positive benefits. Aerobic exercise should be performed at an intensity of > 60% of maximum (either based on HR or VO_2_peak). The addition of resistance training is important for improving muscle function and should be performed at an intensity of > 60% 1-RM at least 2x/week. Exercise, where possible, should be tailored to the comorbidities and the individual’s own goals and capacity. This may require the involvement of an exercise professional, trained in working with clinical populations. The exercise can then be tailored for the patient’s comorbidities and health status. Supervised exercise is likely to maximise results of exercise as workload and intensity can be appropriately managed and changed. However, patients should not be discouraged from exercising on their own or at home. Further resources and evidence are needed to inform of the best practice regarding home-based exercise in KTRs.

### Prehabilitation for transplantation

#### Recommendations

3.13 We suggest that exercise interventions prior to surgery (prehabilitation) may help increase pre-transplant physical activity levels and aid recovery post-transplant [2C]

#### Rationale

Prehabilitation is the process of enhancing patient functional capacity prior to surgery to improve tolerance for the upcoming physiologic stressor [223, 224]. In our meta-analysis, we were unable to identify any studies (RCTs) investigating the role of prehabilitation programmes in KTRs. McAdams-DeMarco et al. [225], in a pre-post pilot study, showed that a prehabilitation programme for KTRs was feasible and that by 2 months of prehabilitation, participants improved their physical activity by 64% (assessed via accelerometery). The prehabilitation programme consisted of supervised cardiovascular and strength exercises, along with stretching and stability training. The authors also reported that among five KTRs who received transplantation during the study period, length of stay was shorter than age-, sex-, and race-matched controls (5 vs. 10 days). These pilot study findings suggest that prehabilitation is feasible in pre-transplant patients and may potentially be a strategy to improve post-transplant outcomes [224].

### Immediate post-transplantation period

#### Recommendations

3.14 We suggest that exercise interventions consisting of intensive physiotherapy and movement encouragement administered immediately post-transplantation i.e. < 1–2 days is not beneficial in increasing recovery or attenuating declines in physical function. However, mobility should be encouraged as per standard care [2C]

#### Rationale

In exercise-based RCT studies [226, 227] involving patients with ‘new’ transplant (i.e. < 2–3 days post-transplant), no additional benefits of exercise were reported, and in the case of Onofre et al. [227], intensive physiotherapy did not attenuate the reductions in exercise capacity or peripheral muscle strength when compared to standard care (which included just simple mobility encouragement). As such, exercise training immediately post-transplantation may not offer any additional benefits above that of standard care. Given the small amount of research into this area, further data is needed to support the use of early intervention post-transplantation.

### Safety and contraindications

#### Recommendations

3.15 We suggest that KTRs avoid traumatic damage to the transplanted kidney and participation in contact sports (e.g., rugby, American football, martial arts, ice hockey, boxing) and/or prolonged extreme exercise (e.g., marathons, Ironman triathlons) must be considered carefully [2C]
3.16 We suggest that KTRs avoid the use of sport-enhancing dietary supplements given the largely unknown potential adverse effects on immune function and potential for unregulated components [2C].

#### Implementing physical activity and exercise guidance

If a KTR is thinking of returning to contact sports, intense prolonged exercise, or taking sports performance-enhancing supplements, they should seek appropriate input from a transplant surgeon, renal and sports medicine clinician, dietician, and an exercise professional.

#### Rationale

Sport, or exercise involving significant contact, may be an appropriate means for an individual to engage in physical activity. Whilst the risk of traumatic damage to transplanted kidneys is low [228], recommendations to participate in contact sports (e.g., rugby, American football, martial arts, ice hockey, boxing) is difficult to support. Combat sports are routinely excluded and the decision to include other activities that could damage or compromise the transplanted organ must be considered carefully [229]. The 2017 Renal Association ‘clinical practice guideline in post-operative care in kidney transplant recipients’, [211] endorsed by the British Transplant Society, encourage participation in sporting events but cautions against participation in sports where a direct blow to the allograft is possible (e.g., kickboxing). Performance in a prolonged extreme environment has been assessed by studying KTRs while trekking in the desert [230]. There were minimal differences between transplant and healthy controls for blood pressure, hydration status, walking velocity, and intensity of physical activity. The selected transplant patients, who had an eGFR > 55 ml/min/1.73m^2^, showed a near-normal physical performance and acclimatisation to the extreme conditions of the desert environment, which suggests that performance of KTRs can be maintained even in challenging environmental conditions. Nonetheless, prolonged, strenuous physically demanding activities, such as marathons, Ironman triathlons etc. challenge many physiological systems and should only be considered by transplanted athletes with a knowledgeable support team that includes an exercise physiologist, an experienced coach, and sports medicine doctor and with the advice of a transplant physician [229]. Whilst there are notable and high-profile cases of KTRs successfully returning to high-intensity sports including professional boxing [231] and rugby, it is important to consider the benefits provided by physical activity, the consequences and safety are also critical outcome measures. Weighing the risk-to-benefit of any activity is an important consideration [229].

Research has shown that the immune system of healthy individuals benefit from regular, moderate physical activity but can be transiently suppressed with prolonged exhaustive exercise [232]. Infection remains a concern in transplant recipients, and, as exercise can be detrimental to the immune system, it should be considered when athletes who are already immunosuppressed are training intensely [233]. There is a lack of knowledge regarding the effects of strenuous exercise on transplant recipients. Königsrainer et al. [234], examined the effects of 81 km of cycling on the immune system of 10 kidney transplant recipients. The authors concluded that transplant recipients showed higher activation of cell metabolism-associated genes but a lack of activation of genes related to immune response when compared with controls immediately post-exercise. These differences between groups reverted to normal one-day post-exercise; it was postulated that the effects might be related to the immunosuppressive medication. Highton et al. [235] found no differences in changes of classical, intermediate, and non-classical monocyte subset proportions, nor the percentage of platelet-derived microparticles that expressed tissue factor (TF+), between groups of non-dialysis patients, healthy controls, and KTRs. As such, moderate exercise did not cause aberrant immune cell activation, supporting its safety from an immunological standpoint.

The use of supplements in sport, with a goal of performance enhancement, is well-established. Fluids for hydration, regeneration and to replenish energy stores are widely promoted and used by athletes and non-athletes. Dietary supplements, nutraceuticals and topical items such as beet juice, β alanine and coconut water are embraced by many athletes looking for competitive advantages. Some have valid physiological effects, most do not, and many contain substances that could have serious contraindications in the management of patients with transplants [229]. Anabolic steroids, stimulants, diuretics that are potentially hazardous to the patient with a transplant can be contained in these products, without appearing on the list of contents and should, therefore, be avoided. Some dietary supplements may potentially interact with the metabolism of immunosuppressive medications if they affect the cytochrome p450 system (e.g., grapefruit extract and tacrolimus). The potential of many dietary supplements to affect immunosuppressive medication drug levels, affect direct or indirect adverse effects of immunosuppressive agents (e.g., hyperkalaemia, renal dysfunction and tacrolimus/cyclosporine) is largely unknown [229]. Some supplements may also contain large amounts of protein which should be considered in those whose whole kidney function is reduced.

In our meta-analysis undertaken for the preparation of these guidelines, the explicit occurrence (or lack of occurrence) in adverse events or injuries were not stated in nine exercise studies. Two studies explicitly stated that no adverse events occurred as part of the intervention [212, 213]. O’Connor et al. [235] reported that, from baseline until 12-months (i.e. encompassing the exercise period reported in Greenwood et al. [212]), 15.4% of patients in the exercise groups were hospitalised, this was compared to 40% of patients in the control arm. They reported no difference in graft rejection rates between groups. No deaths were observed in the study. A higher incidence (30.8%) in of NODAT was seen in both exercising groups compared to 10% in the control group.

### Weight management

#### Recommendations

3.17 We recommend that regular anthropometric measurements should be taken to assess changes in body composition [1B]
3.18 We recommend candidates and KTRs have their body mass (and body mass index, BMI) accurately examined by a healthcare professional at the time of evaluation and while on the waiting list [1B]
3.19 We recommend not excluding candidates based on BMI alone [1B]
3.20 We recommend that potential recipients, not on dialysis, with a BMI > 35 kg/m2 should be actively supported to lose weight via appropriate interventions [1C]
3.21 We recommend that multi-professional weight management services should be available to all KTRs [1C]
3.22 We recommend that post-transplantation an ideal weight should be targeted BMI ≤25 kg/m2) [1B]
3.23 We suggest bariatric surgery can be used to reduce BMI in those with morbid obesity (i.e., >BMI 40 kg/m2)’ [2B]

#### Audit measures

1. Assessment of accurate body mass and BMI via appropriate SOPs (e.g., calibrated scales, no shoes and heavy clothing) at the time of evaluation and while on the waiting list
2. Availability of a multi-professional weight-management services
3. Proportion of patients who are obese (BMI > 30 kg/m^2^) (taken from Baker et al. [211])
4. If feasible, assessment of body composition should be performed

#### Rationale

The impact of obesity on kidney transplant outcomes is complex although study has shown that, *irrespective of BMI*, compared to dialysis treatment patient survival is improved if transplanted [236]. However, in maintenance HD patients, a higher BMI seems to be linked to a survival advantage. Nonetheless, the presence of such obesity survival paradox is unlikely in KTRs since both extremes of pre-transplantation BMI are linked to higher mortality in this population. A meta-analysis of > 300,000 participants by Ahmadi et al. [157] found compared to normal BMI (defined as 18.5 to 24.9), being *underweight* (BMI < 18.5) pre-transplantation [Hazard Ratio (HR): 1.09; 95% CI: 1.02–1.20], *overweight* (BMI 25.0 to 29.9) (HR: 1.07; 95% CI: 1.04–1.12), and *obese* (BMI > 30) (HR: 1.20; 95% CI: 1.14–1.23) were associated with higher mortality. Another meta-analysis conducted by Lafranca et al. [237] found that, from data of 209,000 KTRs, recipients with a higher BMI, graft and patient survival are worse, at least up to 3 years after transplantation.

Previous RA guidelines on the ‘Assessment of the Potential Kidney Transplant Recipient’ (5th edition, 2011) suggested that obese patients (BMI > 30) present ‘technical difficulties’ and are at increased risk of perioperative complications. And that although obesity is not an absolute contraindication to transplantation, individuals with a BMI > 40 kg/m^2^ are less likely to benefit. The ‘Guideline on Kidney Donor and Recipient Evaluation and Perioperative Care’ by the European Renal Best Practice (ERBP) (2014) guideline recommends that candidates with a BMI > 30 kg/m^2^ should lose weight prior to transplant. The Kidney Health Australia – Caring for Australasians with Renal Impairment (KHA-CARI): ‘Recipient Assessment for Transplantation’ and ‘Obesity in renal transplantation’ guidelines (2013) recommend that obesity alone should not preclude a patient from being considered for RT. Furthermore, they state that as a pre-transplant BMI > 40 kg/m^2^ may not be associated with a survival advantage compared to remaining on dialysis, the suitability for transplant should be carefully assessed on an individual basis.

Current 2020 ‘KDIGO Clinical Practice Guideline on the Evaluation and Management of Candidates for Kidney Transplantation’ recommends assessment of all candidates for obesity using either BMI or waist-to-hip criteria. Patients found to be obese or particularly those with class II (BMI 30–34.9 kg/m^2^) or class III obesity (BMI ≥35 kg/m^2^) should be considered for intervention such as dietary counselling or bariatric surgery to achieve a BMI < 30 kg/m^2^. The ERA-EDTA (‘European Renal Best Practice Guideline on kidney donor and recipient evaluation and perioperative care’) reports similar conclusions – they suggest that there is no clear evidence that denying obese patients transplant is in the best interest of the patient regardless of the reduction in post-transplant outcomes. However, they suggest dietary modification and *do not* endorse pharmacologic or surgical weight loss interventions. No UK guidelines on the management of kidney transplant patients (i.e. NICE, RA) discuss the role of bariatric surgery.

A recent meta-analysis [238] of the role of bariatric surgery to achieve transplant in end-stage organ disease patients included 19 studies investigating 288 patients. Findings showed a significant reduction in mean BMI (43.9 to 33.7 kg/m^2^) with 50% of these patients subsequently being listed, and a further 30% transplanted at a mean of 19.9 months post-bariatric surgery. No study described an occurrence of a patient stopping dialysis after weight loss, nor did any study describe an occurrence of pre-dialysis patient who had improvement in kidney function that precluded the need for dialysis. Whilst this suggests bariatric surgery may help patients achieve sufficient weight loss to be eligible for transplant listing, further high-quality studies are needed to investigate the optimal timing and approach of surgical intervention, durability of weight loss in this population, and whether a survival benefit is achieved. KDIGO suggest that studies should investigate the impact of pre-transplant bariatric surgery (e.g., sleeve gastrectomy) on outcomes after kidney transplantation.

Transplantation in patients with a BMI of ≥40 kg/m^2^ should be approached with caution; patients need to understand the increased risk of postoperative complications in this situation. The guideline did not establish a firm BMI cut-off but encourages each transplant program to consider their resources and skills in caring for this population.

Overall, no guideline recommends that obesity (defined on BMI) alone should preclude a patient from being considered for transplantation and that if the transplant surgeon determines that the body composition of the potential recipient does not constitute an increased surgical risk, the patient should be suitable. This is supported by the NICE ‘Renal replacement therapy and conservative management guideline’ [NG107], 2018) which states:

*1.3.7. ‘Do not exclude people from receiving a kidney transplant based on BMI alone’*

Other than relying on BMI alone, assessing body composition, such as skeletal muscle and fat mass separately, may provide greater insight into an individual’s risk of outcome, survival, and post-transplant complications [239]. Techniques such as dual-energy x-ray absorptiometry (DEXA) and multifrequency bioelectrical impedance analysis (MF-BIA) may be valid tools in KTRs [240, 241]. Alternatively, inexpensive and routinely measured surrogate markers such as serum creatinine, waist and hip circumference, or mid-arm muscle circumference can be used [239].

Weight gain post-transplantation frequently appears in the first year after transplant, and it is reported to be a common problem for patients within the first 6 months [157, 222]. Weight gain varies between 6 and 10 kg [242], and the change in mean BMI varies between 2 and 3.8 kg/m^2^ after transplant. Potential factors causing weight gain after kidney transplant are the use of immunosuppressive medications to protect the newly implanted organ and the changes in lifestyle, such as dietary intake and insufficient physical activity [222]. Increased obesity, specifically fat mass, is an important CVD risk factor exacerbating metabolic syndrome and inflammatory status [243], leading to increased mortality and graft failure [157]. Controlling, or limiting, excessive weight gain is a key component of a patient’s post-transplant management [222].

Our meta-analysis showed that structured exercise interventions do not appear to alter body mass or BMI, even in a study targeted at obese patients [218]. However, whilst exercise may attenuate increases in body and/or fat mass in some cases [213], the complex transplantation process may confound any beneficial effects of short-term exercise. Complex long-term interventions encompassing physical activity and dietary behaviour change warrant further investigation.

The Renal Association Clinical Practice Guidelines ‘Post-Operative Care in the Kidney Transplant Recipient’ [211] suggests that KTRs should maintain a BMI ≤25 kg/m^2^.

The KDOQI Clinical Practice Guideline for Nutrition In CKD: 2020 Update [66] states the following in relation to weight and weight management:

- In adults’ post-transplantation, it is reasonable to consider assessing body composition in combination with body weight/BMI at the first visit and to monitor overall nutrition status periodically over time (OPINION)
- In adults with CKD post-transplantation, it is reasonable to use DXA when feasible as it remains the gold standard for measuring body composition despite being influenced by volume status (OPINION).
- In adults’ post-transplantation who are clinically stable, it is reasonable to measure body weight and BMI and to monitor for changes in body weight/BMI and body composition as needed (OPINION) (At least every 3 months in patients post-transplantation)
- In adults with CKD post-transplantation adults, it is reasonable to consider using underweight and overweight or obesity status (based on BMI) as a predictor of higher mortality (OPINION).

### Other lifestyle considerations (smoking, alcohol intake, drug use)

The following is summarised from the Renal Association Clinical Practice Guidelines ‘Post-Operative Care in the Kidney Transplant Recipient’ [211]. There is no evidence to suggest that these recommendations should change.

#### Recommendations

3.24 We recommend that smoking should be strongly discouraged in transplant recipients [1A]
3.25 We suggest alcohol consumption should be within national guidelines [1B]
3.26 We suggest that KTRs avoid all recreational drug use [1B]

#### Audit measures (taken from Baker et al. [211])

1. Proportion of KTRs who smoke
2. Proportion of cigarette smoking KTRs who have been given formal advice or offered help with cessation

#### Rationale

Cigarette smoking is strongly associated with reduced life expectancy, several forms of malignancy, respiratory disease and premature cardiovascular disease in the general population. Whilst the evidence is less comprehensive in KTRs, cigarette smoking is associated with reduced patient survival, malignancy, and increased cardiovascular events [244, 245]. In the general population, various intervention strategies are beneficial in encouraging smoking cessation (nicotine replacements - gum, patch, and inhaled, counselling, and Bupropion) [246]. The long-term benefits of smoking cessation have not been proven in transplant recipients. However, strategies for smoking cessation are safe and likely to produce the same benefits seen in other populations or public health studies. A local strategy should be available and a record made of the advice given and available. Appropriate guidance is available on smoking cessation from NICE: https://cks.nice.org.uk/smoking-cessation and https://cks.nice.org.uk/smoking-cessation#!scenario:1

Alcohol abuse occurs in a small proportion of KTRs though the prevalence and severity of alcohol misuse are difficult to define. Alcohol use within recommended guidelines after transplantation is likely to be safe, whilst alcohol or substance abuse is associated with an increased of premature death [247]. Access to counselling, addiction services and rehabilitation should be available. Appropriate guidance is available on smoking cessation from NICE: https://cks.nice.org.uk/alcohol-problem-drinking#!scenario.

## Supplementary Information

**Additional file 1: Appendix HD1.** Full search strategies for a review of recent systematic reviews and randomised controlled trial data. Physical activity and exercise guidelines for individuals with end-stage kidney disease (ESKD) receiving haemodialysis. **Appendix HD2.** Flow diagram of search results. **Appendix TX1.** Full search strategies for a review of reviews reporting on the importance of physical activity and exercise in renal transplant recipients. **Appendix TX2.** Full search strategies for meta-analysis investigating the evidence for the effect of exercise training interventions in adult kidney transplant recipients. **Appendix TX3.** Flow diagram of systematic search of literature and included studies (until January 2020). **Appendix TX4.** Table of characteristics of included studies. **Appendix TX5.** Forest plots. **Appendix TX6.** Risk of bias summary. **Appendix TX7.** ‘Leave-one-out’ sensitivity analysis. **Appendix TX8.** Funnel plots.

## Data Availability

All data and material used in the production of this guideline can be found within the references.

## Abbreviations

BMI: Body mass index
CKD: Chronic kidney disease
CVD: Cardiovascular disease
DOPPS: Dialysis Outcomes and Practice Patterns Study
ESKD/ESRD: End-stage kidney disease/end-stage renal disease
GPPAQ: General Practice Physical Activity Questionnaire
GREX: Global Renal Exercise
HDL-C: High-density lipoprotein cholesterol
HIIT: High-intensity interval training
HRQoL: Health-related quality of life
KTR: Kidney transplant recipients
LDL-C: Low-density lipoprotein cholesterol
MICT: Moderate-intensity continuous training
ND-CKD: Non-dialysis chronic kidney disease
NICE: National Institute for Health and Care Excellence
PAVS: Physical Activity Vital Sign
QoL: Quality of life
RCT: Randomized control clinical trial
SBP: Systolic blood pressure
USRDS: United States Renal Data System
