# Supplementary material for: Clinical practice guideline exercise and lifestyle in chronic kidney disease
Source: BMC Nephrol. 2022 Feb 22;23:75. doi: 10.1186/s12882-021-02618-1 (PMC8862368; doi:10.1186/s12882-021-02618-1)
Supplement: Supplementary file 1 — Additional file 1: Appendix HD1. Full search strategies for a review of recent systematic reviews and randomised controlled trial data. Physical activity and exercise guidelines for individuals with end-stage kidney disease (ESKD) receiving haemodialysis. Appendix HD2. Flow diagram of search results. Appendix TX1. Full search strategies for a review of reviews reporting on the importance of physical activity and exercise in renal transplant recipients. Appendix TX2. Full search strategies for meta-analysis investigating the evidence for the effect of exercise training interventions in adult kidney transplant recipients. Appendix TX3. Flow diagram of systematic search of literature and included studies (until January 2020). Appendix TX4. Table of characteristics of included studies. Appendix TX5. Forest plots. Appendix TX6. Risk of bias summary. Appendix TX7. ‘Leave-one-out’ sensitivity analysis. Appendix TX8. Funnel plots. [file 12882_2021_2618_MOESM1_ESM.docx]

# Appendices – Non-dialysis CKD (stages 1-5)

Appendix ND-CKD1.

**Example search strategies for a review of recent systematic reviews and randomised controlled trial data. Physical activity and exercise guidelines for individuals with non-dialysis CKD (stages 1-5)**

a) *Final Medline search (Mortality, hospitalisations and disease progression)*

(("Renal Insufficiency, Chronic"[Mesh]) OR ("Kidney Failure, Chronic"[Mesh]) OR ("Diabetic Nephropathies"[Mesh]) OR ("Nephrotic syndrome, idiopathic, steroid-resistant" [Supplementary Concept]) OR ("Glomerulonephritis"[Mesh]) OR ("Chronic Kidney Disease"[Title/Abstract])) AND (("Exercise"[Mesh]) OR ("Exercise therapy"[Mesh]) OR ("Exercise Movement Techniques"[Mesh]) OR ("Rehabilitation"[Mesh]) OR ("Physical Fitness"[Mesh]) OR ("exercise"[Title/Abstract]) OR ("kinesiotherapy"[Title/Abstract]) OR ("physical rehabilitation"[Title/Abstract]) OR ("physical training"[Title/Abstract]) OR ("physical activity"[Title/Abstract]) OR ("physical fitness"[Title/Abstract]) OR ("resistance training"[Title/Abstract]) OR ("resistance exercise"[Title/Abstract]) OR ("resistance program"[Title/Abstract]) OR ("strength program"[Title/Abstract]) OR ("strength exercise"[Title/Abstract]) OR ("strength training"[Title/Abstract]) OR ("aerobic training"[Title/Abstract]) OR ("aerobic program"[Title/Abstract]) OR ("aerobic exercise"[Title/Abstract])) AND ( ("morbidity" [Title/Abstract]) OR ("mortality" [Title/Abstract]) OR ("cardiovascular events" [Title/Abstract]) OR ("hospitalisations" [Title/Abstract]) OR ("creatinine" [Title/Abstract]) OR ("eGFR" [Title/Abstract]) OR ("proteinuria" [Title/Abstract]) OR ("albuminuria" [Title/Abstract]) OR ("renal biomarker" [Title/Abstract]) OR ("leucocyturia" [Title/Abstract]) OR ("haematuria" [Title/Abstract])) Filters: Clinical Trial, Meta-Analysis, Randomized Controlled Trial, Humans, English

b) *Final Medline search (Exercise capacity, muscle mass and function)*

(("Renal Insufficiency, Chronic"[Mesh]) OR ("Kidney Failure, Chronic"[Mesh]) OR ("Diabetic Nephropathies"[Mesh]) OR ("Nephrotic syndrome, idiopathic, steroid-resistant" [Supplementary Concept]) OR ("Glomerulonephritis"[Mesh]) OR (“Chronic Kidney Disease”[Title/Abstract])) AND ((“Exercise”[Mesh]) OR (“Exercise therapy”[Mesh]) OR (“Exercise Movement Techniques”[Mesh]) OR (“Rehabilitation”[Mesh]) OR (“Physical Fitness”[Mesh]) OR (“exercise”[Title/Abstract]) OR (“kinesiotherapy”[Title/Abstract]) OR (“physical rehabilitation”[Title/Abstract]) OR (“physical training”[Title/Abstract]) OR (“physical activity”[Title/Abstract]) OR (“physical fitness”[Title/Abstract]) OR (“resistance training”[Title/Abstract]) OR (“resistance exercise”[Title/Abstract]) OR (“resistance program”[Title/Abstract]) OR (“strength program”[Title/Abstract]) OR (“strength exercise”[Title/Abstract]) OR (“strength training”[Title/Abstract]) OR (“aerobic training”[Title/Abstract]) OR (“aerobic program”[Title/Abstract]) OR (“aerobic exercise”[Title/Abstract])) AND  ( (Topic-specific [Mesh terms]) OR (Topic-specific [Title/Abstract]) )

((renal[Title/Abstract] OR kidney[Title/Abstract]) AND (exercise[Title/Abstract] OR physical activity[Title/Abstract] OR resistance[Title/Abstract] OR strength[Title/Abstract] OR kinesiotherapy[Title/Abstract] OR rehabilitation[Title/Abstract] OR training[Title/Abstract] OR fitness[Title/Abstract] OR aerobic[Title/Abstract])) AND (trial[Title/Abstract] OR intervention[Title/Abstract] OR randomi*ed[Title/Abstract] OR RCT[Title/Abstract])

and limit from 2014- 12 February 2020

c) *Final Medline search (Metabolic risk factors)*

(("Renal Insufficiency, Chronic"[Mesh]) OR (chronic kidney disease) OR CKD) AND( "Exercise"[Mesh] OR"Rehabilitation"[Mesh] OR "exercise therapy"[MeSH] OR "Exercise Movement Techniques"[Mesh] OR exercise therapy[Title/Abstract] OR physical training[Title/Abstract] OR physical activity[Title/Abstract] OR physical fitness[Title/Abstract] OR resistance training[Title/Abstract] OR resistance exercise[Title/Abstract] OR resistance program[Title/Abstract] OR strength program[Title/Abstract] OR strength exercise[Title/Abstract] OR strength training[Title/Abstract] OR aerobic training[Title/Abstract] OR aerobic program[Title/Abstract] OR aerobic exercise[Title/Abstract] OR tai chi[Title/Abstract] OR Exercise therapy[Title/Abstract]))

d) *Final Medline search (Cognitive function)*

**(**("Renal Insufficiency, Chronic"[Mesh]) OR ( "Kidney Failure, Chronic"[Mesh]) OR  ("Diabetic Nephropathies"[Mesh] ) OR ("Nephrotic syndrome, idiopathic, steroid-resistant" [Supplementary Concept]) OR ("Glomerulonephritis"[Mesh]))

AND (("Cognition"[Mesh]) OR ("Cognition Disorders"[Mesh]) OR ("Mental Status and Dementia Tests"[Mesh])) AND (("Exercise"[Mesh]) OR ("Physical Fitness"[Mesh]) OR ("Exercise Therapy"[Mesh]))

Appendix ND-CKD2.

**Flow diagrams of systematic search of literature and included studies (until July 2020)**

**a)**

Records identified through database searching (MEDLINE) (PubMed)
(n= 1083)

Additional records identified through other sources (manual search through reference lists)

(n= 0)

Records after duplicates excluded

(n= 1083)

Records screened

(n= 1083)

**Records excluded**

Not eligible (n= 949)

**Full text articles excluded, with reasons (110)**

Article type (n= 12)

Study design (single-arm or non-randomised) (n= 8)

No relevant outcome measure (n= 68)

Intervention (n= 22)

Duplicate study (n= 2)

Full-text articles assessed for eligibility

(n= 134)

Trials reviewed for qualitative synthesis in Guidelines:

Morbidity (n= 4)

Mortality (n= 2)

Cardiovascular events (n= 0)

Hospitalisations (n= 1)

Creatinine (n= 2)

eGFR (n= 18)

albuminuria/proteinuria (n= 8)

haematuria/leucocyturia (n= 0)

renal biomarker (n= 3)

**b)**

Records identified through database searching (MEDLINE, PubMed, Web of Science) (n= 2301)

Additional records identified through other sources (manual search through SR reference lists)

(n= 21)

Records after duplicates excluded

(n= 1429)

Title/ abstracts screened

(n= 1429)

**Records excluded**

Not eligible (n= 1391)

**Full text articles excluded, with reasons (32)**

Article type (n= 8)

Population (n= 3)

Intervention (n= 12)

Outcome (n= 8)

Duplicate publication (n= 1)

Full-text articles assessed for eligibility

(n= 40)

Trials reviewed for qualitative synthesis in Guidelines:

Functional limitations (n= 6)

Wellbeing (n= 1)

KD-specific symptoms (n= 1)

**c)**

Additional records identified through other sources (manual search through SR reference lists)

(n= 0)

Records identified through database searching (MEDLINE, PubMed, Web of Science) (n= 1599)

Records after duplicates excluded

(n= 1599)

**Records excluded**

Not eligible (n= 1548)

Title/ abstracts screened

(n= 1599)

**Full text articles excluded, with reasons (7)**

Article type (n= 1)

Study type (n= 3)

Intervention (n= 1)

Outcome measure (n= 1)

Duplicate publication (n= 1)

Full-text articles assessed for eligibility

(n= 51)

Trials reviewed for qualitative synthesis in Guidelines:

Blood Pressure (n= 21)

Lipids (n= 17)

Inflammation (n= 16)

Oxidative Stress (n= 3)

Glycaemic control (n= 8)

Albumin (n= 5)

**d)**

Additional records identified through other sources (manual search through reference lists)

(n= 30)

Records identified through database searching (MEDLINE) (PubMed)
(n= 155)

Records after duplicates excluded

(n= 150)

**Records excluded**

Not eligible (n= 124)

Records screened

(n= 150)

Full-text articles assessed for eligibility

(n= 26)

**Full text articles excluded, with reasons (0)**

Trials reviewed for qualitative synthesis in Guidelines:

Body composition (n= 24)

Cognitive function (n= 2)

# Appendices - Haemodialysis

Appendix HD1.

**Full search strategies for a review of recent systematic reviews and randomised controlled trial data. Physical activity and exercise guidelines for individuals with end-stage kidney disease (ESKD) receiving haemodialysis**

The following systematic review search was used to identify systematic reviews (and meta-analyses) or randomised controlled trials or that may be relevant for the Guideline document.

| **Objective** | |
| --- | --- |
| To perform a search for clinical trials and systematic reviews (and meta analyses), that will inform the writing of the new Renal Association Guidelines for physical activity and lifestyle in the haemodialysis population. | |
| **Methods** | |
| Study eligibility criteria | |
| **Types of trial or review:** | Trials were considered for inclusion if they were randomised controlled trials that involved allocation of participants at an individual or cluster level, or via quasi-randomised method. Systematic reviews alone or systematic reviews with meta-analysis of these trials were also considered. |
| **Population:** | *Uptake*: Adults (>18 years) with end-stage kidney disease who are receiving prevalent (for at least 3 months) haemodialysis. |
| **Intervention:** | Any physical activity, exercise, lifestyle, weight loss or smoking cessation intervention aimed at improving outcomes in participants with end-stage kidney disease receiving prevalent haemodialysis as a form of renal replacement therapy  Comparison: Any concurrent control group who is receiving usual care (i.e. not receiving the physical activity, exercise or lifestyle, weight loss or smoking cessation intervention). |
| **Outcomes** | *Primary*:   - Any clinical outcome - Patient reported outcome |

| **Data collection** | |
| --- | --- |
| Search methods for identification of trials, national guidelines and clinical practice recommendations | Searches of systematic review databases was conducted; Cochrane and PROSPERO. MEDLINE was searched which includes the National Centre for Biotechnology Information (NCBI) PubMed. |
| Search terms  (example for Medline and CINAHL provided) | \| **Search term** \| **Field**; **1** \| Renal insufficiency \| MH (explode); **2** \| Renal insufficiency \| TX; **3** \| Renal insufficiency, chronic \| MH (explode); **4** \| Renal insufficiency, chronic \| TX; **5** \| Renal Dialysis \| MH; **6** \| Renal dialysis \| TX; **7** \| Kidney failure, chronic \| MH; **8** \| Kidney failure, chronic \| TX; **9** \| Renal Replacement therapy \| MH; **10** \| Renal Replacement therapy \| TX; **11** \| Haemodialysis \| TX; **12** \| Hemodialysis \| TX; **13** \| Dialysis \| TX; **14** \| ESRD \| TX; **15** \| ESKD \| TX; **16** \| kidney disease adj2 (end-stage OR end stage OR chronic) \| TX; **17** \| renal disease adj2 (end-stage OR end stage OR chronic) \| TX; **18** \| kidney failure adj2 (end-stage OR end stage OR chronic) \| TX; **19** \| Renal replacement therapy \| TX; **20** \| renal failure adj2 (end-stage OR end stage OR chronic) \| TX; **21** \| 1 OR 2 OR 3 OR 4 OR 5 OR 6 OR 7 OR 8 OR 9 OR 10 OR 11 OR 12 OR 13 OR 14 OR 15 OR 16 OR 17 OR 18 OR 19 OR 20 \|; **22** \| Clinical Trial \| MH; **23** \| Randomized Controlled Trials as Topic \| MH; **24** \| Randomi?ed Controlled Trials \| TX; **25** \| Random Allocation \| MH; **26** \| Random Allocation \| TX; **27** \| Meta-Analysis \| MH; **28** \| Meta-Analysis \| TX; **29** \| Systematic Review \| MH; **30** \| Systematic Review \| TX; **31** \| 22 OR 23 OR 24 OR 25 OR 26 OR 27 OR 28 OR 29 OR 30 \|; **32** \| Exercise Therapy \| MH (explode); **33** \| Exercise Therapy \| TX; **34** \| Healthy Lifestyle \| MH (explode); **35** \| Healthy Lifestyle \| TX; **36** \| Life Style \| MH (explode); **37** \| Life Style \| TX; **38** \| “Physical and Rehabilitation Medicine” \| MH; **39** \| “Physical and Rehabilitation Medicine” \| TX; **40** \| Intradialytic \| TX; **41** \| Intra-dialytic \| TX; **42** \| Intradialytic exercise \| TX; **43** \| Intra-dialytic exercise \| TX; **44** \| Exercise training \| TX; **45** \| Exercise programme \| TX; **46** \| Aerobic Training \| TX; **47** \| Aerobic adj2 Training \| TX; **48** \| Aerobic adj2 Intervention \| TX; **49** \| Resistance training \| TX; **50** \| Lifestyle Intervention \| TX; **51** \| Lifestyle adj2 Intervention \| TX; **52** \| Weight Loss \| MH; **53** \| Weight Loss \| TX; **54** \| Diet, Reducing \| TX; **55** \| Weight Reduction Programs \| MH (explode); **56** \| Weight Reduction Programs \| TX; **57** \| Weight adj2 Change \| TX; **58** \| Weight Loss Program* \| TX; **59** \| Weight program* \| TX; **60** \| Weight adj2 Program* \| TX; **61** \| Weight Control* \| TX; **62** \| Weight Train* \| TX; **63** \| Weight Reduc* \| TX; **64** \| Smoking* \| MH (explode); **65** \| Smoking \| TX; **66** \| Smoking Cessation* \| MH (explode); **67** \| Smoking Cessation \| TX; **68** \| Smoking Reduction* \| MH; **69** \| Smoking Reduction \| TX; **70** \| Tobacco \| TX; **71** \| Cessation \| TX; **72** \| Exercise \| TX; **73** \| 32 or 33 or 34 or 35 or 36 or 37 or 38 or 39 or 40 or 41 or 42 or 43 or 44 or 45 or 46 or 47 or 48 or 49 or 50 or 51 or 52 or 53 or 54 or 55 or 56 or 57 or 58 or 59 OR 60 OR 61 OR 62 OR 63 OR 64 OR 65 OR 66 OR 67 OR 68 OR 69 OR 70 OR 71 \|; **74** \| 21 AND 31 AND 73 \| |

| **Data extraction** | |
| --- | --- |
| Data extraction, management and synthesis. | Titles and abstracts were uploaded into the Endnote referencing software and screened by DM in line with the inclusion criteria. Full text papers will be requested and assessed by DM when trials or systematic reviews were not excluded based on title or abstract. Any trials or systematic reviews that were deemed unclear for inclusion were discussed and checked with JOB and MGB.  Trials and systematic reviewers were included if they provide evidence to inform guidelines as to physical activity recommendations outside of haemodialysis, intradialytic exercise (including safety and implementation), evidence and guidance for weight loss or maintenance, and smoking cessation in the haemodialysis population. Data was synthesised narratively in guideline format. Using this format, statements regarding the evidence were made and these were graded on strength using the Modified GRADE system (1) |

Appendix HD2.

**Flow diagram of search results**

Records identified through database searching (MEDLINE) (PubMed)
(n=941)

Records excluded
(n=807)

Records screened

(n=941)

Full text articles excluded, with reasons
CKD patients not receiving dialysis (n=6)

Full-text articles assessed for eligibility

(n = 131)

Trials reviewed for qualitative synthesis in Guideline

Exercise/Physical activity reviews (n = 59)

Exercise/Physical activity trials (n=58)

Weight loss reviews (n=4)

Weight loss trials (n=0)

Smoking reviews (n=4)

Smoking trials (n=0)

# Appendices - Transplantation

Appendix TX1.

**Full search strategies for a review of reviews reporting on the importance of physical activity and exercise in renal transplant recipients**

**MEDLINE (1966-present day)**

**Final search details:**

("review"[Publication Type] OR "review literature as topic"[MeSH Terms] OR "review"[All Fields]) AND (((("exercise"[MeSH Terms] OR "exercise"[All Fields]) OR ("exercise therapy"[MeSH Terms] OR ("exercise"[All Fields] AND "therapy"[All Fields]) OR "exercise therapy"[All Fields])) OR ("exercise"[MeSH Terms] OR "exercise"[All Fields] OR ("physical"[All Fields] AND "activity"[All Fields]) OR "physical activity"[All Fields])) AND ((("kidney transplantation"[MeSH Terms] OR ("kidney"[All Fields] AND "transplantation"[All Fields]) OR "kidney transplantation"[All Fields] OR ("renal"[All Fields] AND "transplantation"[All Fields]) OR "renal transplantation"[All Fields]) OR ("kidney transplantation"[MeSH Terms] OR ("kidney"[All Fields] AND "transplantation"[All Fields]) OR "kidney transplantation"[All Fields])) OR ("transplant recipients"[MeSH Terms] OR ("transplant"[All Fields] AND "recipients"[All Fields]) OR "transplant recipients"[All Fields])))

Latest search date: **15th January 2020**

**Strategy structure and breakdown of search returns:**

| **Search term** | **Items returned** |
| --- | --- |
| 1. Renal Transplantation | 140810 |
| 2. Kidney Transplantation (MESH) | 126919 |
| 3. Transplant Recipients (MESH) | 66591 |
| 1. 1 OR 2 OR 3   ((Renal Transplantation) OR Kidney Transplantation) OR Transplant Recipients | 175149 |
| 4. Exercise (MESH) | 414127 |
| 5. Exercise therapy (MESH) | 116101 |
| 6. Physical activity | 536949 |
| 2. 5 OR 6 OR 7   ((Exercise) OR Exercise therapy) OR Physical activity | 537454 |
| 3. 4 AND 8   ((((Exercise) OR Exercise therapy) OR Physical activity)) AND (((Renal Transplantation) OR Kidney Transplantation) OR Transplant Recipients) | 1440 |
| 7. Review | 3214971 |
| 4. 9 and 10   (Review) AND (((((Exercise) OR Exercise therapy) OR Physical activity)) AND (((Renal Transplantation) OR Kidney Transplantation) OR Transplant Recipients)) | 281 |
| 8. Limited search to last 5 years | 87 |

Search retuned: #115 studies

#102 studies were removed

| **Reason** | **Number excluded after abstract review** |
| --- | --- |
| Dialysis patients | 4 |
| Non-dialysis CKD | 2 |
| Non-kidney disease | 43 |
| Protocol paper | 1 |
| Non-exercise/physical activity topic | 24 |

| Remaining total based on initial review of abstract and full text sought | #14 |
| --- | --- |
| Removed after full text review | 0 |
| Total left for review and analysis | #14 |

Appendix TX2.

**Full search strategies for meta-analysis investigating the evidence for the effect of exercise training interventions in adult kidney transplant recipients**

**MEDLINE (1966-present day)**

**Final search details:**

(((("exercise"[MeSH Terms] OR "exercise"[All Fields]) OR ("exercise therapy"[MeSH Terms] OR ("exercise"[All Fields] AND "therapy"[All Fields]) OR "exercise therapy"[All Fields])) OR ("exercise"[MeSH Terms] OR "exercise"[All Fields] OR ("physical"[All Fields] AND "activity"[All Fields]) OR "physical activity"[All Fields])) AND ((("kidney transplantation"[MeSH Terms] OR ("kidney"[All Fields] AND "transplantation"[All Fields]) OR "kidney transplantation"[All Fields] OR ("renal"[All Fields] AND "transplantation"[All Fields]) OR "renal transplantation"[All Fields]) OR ("kidney transplantation"[MeSH Terms] OR ("kidney"[All Fields] AND "transplantation"[All Fields]) OR "kidney transplantation"[All Fields])) OR ("transplant recipients"[MeSH Terms] OR ("transplant"[All Fields] AND "recipients"[All Fields]) OR "transplant recipients"[All Fields]))) AND ("randomized controlled trial"[Publication Type] OR "randomized controlled trials as topic"[MeSH Terms] OR "randomized controlled trial"[All Fields] OR "randomised controlled trial"[All Fields])

Latest search date: **16th January 2020**

**Strategy structure and breakdown of search returns:**

| **Search term** | **Items returned** |
| --- | --- |
| 1. Renal Transplantation | #153280 |
| 2. Kidney Transplantation (MESH) | #134638 |
| 3. Transplant Recipients (MESH) | #65016 |
| 4. 1 OR 2 OR 3   ((Renal Transplantation) OR Kidney Transplantation) OR Transplant Recipients | #186339 |
| 5. Exercise (MESH) | #447282 |
| 6. Exercise therapy (MESH) | #101084 |
| 7. Physical activity | #537855 |
| 8. 5 OR 6 OR 7   ((Exercise) OR Exercise therapy) OR Physical activity | #564099 |
| 9. 4 AND 8   ((((Exercise) OR Exercise therapy) OR Physical activity)) AND (((Renal Transplantation) OR Kidney Transplantation) OR Transplant Recipients) | #1671 |
| 10. Randomi?ed controlled* trial (MESH) | #646726 |
| 11. 9 AND 10   ((((((Exercise) OR Exercise therapy) OR Physical activity)) AND (((Renal Transplantation) OR Kidney Transplantation) OR Transplant Recipients))) AND Randomized controlled trial | #115 |

Search retuned: #115 studies

#102 studies were removed

| **Reason** | **Number excluded after abstract review** |
| --- | --- |
| Review paper | #4 |
| Dialysis | #20 |
| Non-dialysis CKD | #5 |
| Non-KD | #55 |
| Protocol paper | #4 |
| Non-exercise intervention | #14 |

| Remaining total based on initial review of abstract and full text sought | #13 |
| --- | --- |
| Removed after full text review | #4 (1x only exercise counselling, 1x protocol paper, 1x article in Czech, 1x mainly diet intervention with physical activity advice) |
| Added from hand-searching reference lists | #4 |
| Total left for review and analysis | #13 |

**Cochrane Central Register of Controlled Trials (includes EMBASE, ICTRP)**

**Final search details:**

‘exercise therapy’ ‘physical activity’ ‘kidney transplantation’ ‘renal transplant recipients’ ‘randomized controlled trial’

Latest search date: **16th January 2020**

Search retuned: #4 studies

#4 studies were removed

| **Reason** | **Number excluded after abstract review** |
| --- | --- |
| Duplicate | #1 |
| Protocol paper | #2 |
| Conference abstract | #1 |

Remaining total based on initial review of abstract and full text sought #0

Appendix TX3.

**Flow diagram of systematic search of literature and included studies (until January 2020)**

#4 added following review of reference lists

#10 of studies included in quantitative synthesis (meta-analysis)

#13 of studies included in qualitative synthesis

#13 of full-text articles assessed for eligibility

#118 of records screened

#118 of records after duplicates removed

**#4 of full-text articles excluded** (Only counselling intervention, #2; Protocol paper, #1; Article in Czech, #1)

**#105 of records excluded** (review paper, #4; Study of dialysis patients, #20; Study in non-dialysis patients, #5; Study in non kidney disease patients, #55; Protocol paper, #6; Non-exercise/physical activity intervention, #14; Abstract, #1)

**#0 of additional records identified through other sources**

**#119 of records identified through database searching** (Pubmed (MEDLINE), #115, CCRCT (including EMBASE, ICTRP), #4)

Appendix TX4.

**Table of characteristics of included studies**

| **Reference and country of origin** | **Mean age, years** | **Mean duration after transplant, years** | **Sample size, n^a^** | **Duration** | **Exercise intervention summary** | **Control group (comparator)** | **Summary of main outcomes (difference from control)** | **Adverse events** |
| --- | --- | --- | --- | --- | --- | --- | --- | --- |
| Onofre et al.  Brazil | Ex, 37.0  Con, 35.6 | Exercise began 1 day post-operative | Ex, 30  Con, 33 | Until discharge (mean in Ex group was 7.1 days) | Daily post-operative supervised physiotherapy for 30 minutes (upper limb movement, walking, step-ups). Resistance training introduced from Day 2 | Standard care (including daily visits from physio and mobility encouragement) | ↔6MWT  ↑Respiratory muscle strength  ↑Maximum expiratory pressure  ↔Upper limb strength  ↔Lower limb strength | Not stated |
|  |  |  |  |  |  |  |  |  |
| O'Connor et al. UK | Ex^b^, 54.3  Con, 49.5 | Ex^b^, 29.3 weeks  Con, 27.7 weeks | Ex^b^, 40  Con, 20 | 9 month self-managed physical activity | Encouraged to engage with community exercise pathways following end of involvement in 12-week exercise programme | Usual care (no specific exercise guidance other than at routine appointments) | ↓PWV  ↔VO_2_peak  ↔SBP  ↔DBP | Described in table footer^c^ |
|  |  |  |  |  |  |  |  |  |
| Greenwood et al. UK | Ex^b^, 54.3  Con, 49.5 | Ex^b^, 29.3 weeks  Con, 27.7 weeks | Ex^b^, 40  Con, 20 | 12 weeks | Aerobic (AT) or resistance training (RT) groups 3x/week. AT consisted of static machines, 80% HR maximum, RPE 13-15. Training duration was progressed to 60 minute per session. RT intensity set at 80% 1-RM for both upper and lower body exercises. Frequency built to 3 sets of 8-10 repetitions | Seen in routine clinic and not referred to any exercise intervention pathways | ↑PWV  ↑VO_2_peak  ↑ Lower limb strength (RT group only)  ↑STS-60 (RT group only)  ↑DASI  ↔SF-36  ↔eGFR  ↔BMI  ↑ Weight  ↓Resting HR (RT group only)  ↔SBP  ↓DBP (RT group only)  ↔CRP  ↔IL-6  ↔TNF-a  ↔TNFR-1  ↔TNFR-2  ↔Fetuin | No adverse events specifically reported |
|  |  |  |  |  |  |  |  |  |
| Tzvetanov et al. USA | Ex, 46  Con, 45 | Ex, 8.6 months  Con, 10.9 months | Ex, 9  Con, 8 | 12 months | ‘The GH Method’ - consists of individual physical training (one-to-one  sessions with a GH coach) using low-impact, low-repetition,  resistance-based weight training with two 1-hour sessions each week | Standard of care  which includes dietary and exercise counselling  by the transplant nutritionist at the time of transplantation  and additional dietary and exercise counselling at clinic visits. | ↔BMI  ↔Lean mass  ↔Fat mass  ↔SBP  ↔DBP  ↓PWV  ↔eGFR  ↔Creatinine  ↔Lipoproteins  ↔Triglycerides, ↔Fasting blood glucose  ↔Haemoglobin  ↑SF-36 | Not stated |
| Riess et al. Canada | Ex, 56.9  Con, 52.4 | Ex, 6.4 years  Con, 9.1 years | Ex, 16  Con, 15 | 12 weeks | Aerobic training 3x/week at 60-80% VO_2_max for 30-60 minutes on bike or treadmill. Strength 2x/week. Only lower body exercises included. 2 sets of 10-15 repetitions | Not provided with exercise guidelines and continued with their usual activities | ↑ VO_2_peak  ↔Watts  ↑ Cardiac output  ↑ Maximum HR  ↔Stroke volume  ↔Arterial compliance  ↔SBP  ↔DBP  ↑ Lower body strength  ↔Lean mass  ↔CVD risk score  ↑ SF-36 | Not stated |
|  |  |  |  |  |  |  |  |  |
| Kouidi et al. Greece | Ex, 52.1  Con, 52.6 | Ex, 22 months  Con, 22.1 months | Ex, 12  Con, 12 | 6 months | Supervised 4x/week for 60-90 minutes, mainly aerobic-based training (set at 50–75% VO_2_peak or 65–85% maximum HR). Included strengthening exercises for upper and lower body as well as abdominal muscles. 2-3 sets of 10-12 repetitions. | Sedentary patient control group who were instructed to refrain from  exercising during the study period | ↔Resting HR  ↔SBP  ↔DBP  ↑Maximum HR  ↔VEpeak  ↑VO_2_peak  ↑HRV markers | Not stated |
| Juskowa et al. Poland | Ex, 43.8  Con, 46.1 | Ex, 22.3 days  Con, 30.8 days | Ex, 32  Con, 37 | 4-5 weeks post-transplant | Supervised every other day for 30 minutess (on other days unsupervised). Exercise included strengthening the muscles  of the upper and lower body as well as abdominal muscles (care of the surgical wound). Breathing, coordination,  isometric, and relaxation exercises also included | Standard care control group – standard care not described | ↔Haemoglobin  ↔Fibrinogen  ↔Creatinine  ↔Glucose  ↔t-Hcy  ↔Folate  ↔Vit B12  ↔IL-18  ↔Total protein  ↔Albumin  ↔Total cholesterol  ↔LDL  ↔Triglycerides  ↔HDL | Not stated |
|  |  |  |  |  |  |  |  |  |
| Painter et al. (2002) USA | Ex, 39.7  Con, 43.7 | Not stated | Ex, 54  Con, 43 | 12 months | Home-based aerobic exercise that consisted of walking/cycling 4x/week for at least 30 minutes (intensity set at 60-85% maximum HR, progressed up to 75-80%) | Usual care group | ↑VO_2_peak  ↑Lower body strength  ↔Body mass  ↔BMI,  ↔Fat mass,  ↔lLean mass, ↔BMD (total)  ↑SF-36  ↔Creatinine  ↔BUN  ↑Haematocrit  ↑Haemoglobin | Not stated |
|  |  |  |  |  |  |  |  |  |
| Painter et al. (2003) USA | Ex, 39.7  Con, 43.7 | Not stated | Ex, 51  Con, 45 | 12 months | See above | Usual care group | ↔Total cholesterol  ↑HDL  ↔SBP  ↔DPB  ↔CVD risk score  ↔BMI  ↑METS | Not stated |
|  |  |  |  |  |  |  |  |  |
| Karelis et al. Canada | Ex, 45.3  Con, 39.4 | Not stated | Ex, 12  Con, 12 | 16 weeks | Supervised resistance training 3x/week in a gym and 2x/week at home. Sessions lasted between 45-60 minutes. Supervised sessions included leg press, chest press, arm curls at 80% 1-RM. Home-based resistance training was performed using elastic bands. | Control group – instructed not to perform any structured  exercises | ↑WHO-5 well-being score  ↔Body mass  ↔Fat mass  ↔Lean mass  ↔VO_2_peak  ↔Strength  ↔Triglycerides  ↔Total cholesterol  ↔HDL  ↔LDL  ↔Glucose  ↔ A1C  ↔HOMA | No injuries or adverse events reported |
| Korabiewska et al. Poland | Ex, 42.6  Con, 43.8 | Not stated | Ex, n=35  Con, n=32 | 12 months | First 6 months involved upper/lower strength training, breathing and coordination exercises for 2/3x/week for 20-35 minutes. In the second 6 months, walking for 10-20 minutes 2/3x/week was included | Control group – underwent “periodic examinations” | ↔ Creatinine  ↔ Haemoglobin  ↔ Albumin  ↔ LDH  ↔ PEF  ↔ Upper limb strength  ↔ Joint extension | Not stated |
|  |  |  |  |  |  |  |  |  |
| Pooranfar et al. Iran | Only total reported  36.3 yrs | Not stated | Ex, 29  Con, 15 | 10 weeks | 60-90 minutes session, 3x/week. Combination of aerobic cycling, treadmill (set at 40-70% maximum HR) and free weights using a circuit format | “The control group experienced no  regular physical exercise” | ↑Sleep quantity  ↑Sleep quality  ↓LDL  ↔HDL  ↓Total cholesterol  ↓Triglycerides | Not stated |
|  |  |  |  |  |  |  |  |  |
| Eatemadololama et al. Iran | Ex, 27.4  Con, 36.0 | Not stated | Ex, 12  Con, 12 | 12 weeks | Supervised 2x/week upper and lower body resistance training, 1 set of 10-15 repetitions at 50% 1RM. Training also included 10 minutes cycling | “Control group without any intervention” | ↑BMD (femur)  ↔BMD (lumbar spine) | Not stated |

Ex = Exercise group; Con = Control; 6MWT = 6-minute walk test; PWV = Pulse wave velocity; SBP = Systolic blood pressure DBP = Diastolic blood pressure HR = Heart rate 1-RM = 1-repeitition maximum; STS-60 = Sit-to-stand-60 test; DASI = Duke Activity Status Index; eGFR = Estimated glomerular filtration rate; BMI = Body mass index; CRP = C-reactive protein; IL-6 = Interleukin-6; TNF = Tumor necrosis factor (α, alpha, R, receptor); SF-36 = Short-form 36 questionnaire; CVD = Cardiovascular disease; VE = Ventilation equivalent; HRV = Heart rate variability; METS = Metabolic equivalent; A1C = Glycated haemoglobin; HOMA = Homeostatic model assessment; LDH = Lactate dehydrogenase; PEF = Peak expiratory flow; BMD = Bone mineral density; ^a^ total sample size of those randomised; ^b^ combined age and transplant durations of the exercise groups as was included in the meta-analysis; ^c^ 15.4% patients hospitalised in Ex group, compared to 40% in control group (from baseline) No difference in rejection rates between groups, no deaths. Higher incidence (30.8%) in Ex groups of NODAT compared to 10% in control

Appendix TX5.

**Forest plots**

*Forest plot comparing exercise with control on changes in VO_2_peak. Post-intervention VO2peak values in Koudi et al. were calculated using the baseline values and mean change scores reported. The SD was estimated from the 95% CI provided.*

*Forest plot comparing exercise with control on changes in body mass (above) and BMI (below). Changes in BMI in Tzvetanov et al. were calculated using mean change and baseline values.*

*Forest plot comparing exercise with control on changes in maximum (above) and resting (below) heart rate (HR). Post-intervention HR values in Kouidi et al. were calculated using the baseline values and mean change scores reported. The SD was estimated from the 95% CI provided.*

*Forest plot comparing exercise with control on changes in creatinine*

*Forest plot comparing exercise with control on changes in systolic (above) and diastolic (below) blood pressure. Data from Riess et al. inputted as change from baseline*

*Forest plot comparing exercise with control on changes in haemoglobin*

*Forest plot comparing exercise with control on changes in lipid profile (HDL, LDL, triglycerides, and fasting glucose)*

Appendix TX6.

**Risk of bias summary**

Appendix TX7.

**‘Leave-one-out’ sensitivity analysis**

We employed a ‘leave-one-out’ sensitivity analysis to all outcomes that were included in the quantitative analysis. Leave-one-out meta-analysis involves performing a meta-analysis on each subset of the studies obtained by leaving out exactly one study. This shows how each individual study affects the overall estimate of the rest of the studies. Data for ‘leave-one-out’ Z and P values presented in the same study order as figures in main manuscript.

| **Outcome** | **All studies** | | **‘leave-one-out’** | | **Conclusion** |
| --- | --- | --- | --- | --- | --- |
|  | **Z** | **P** | **Z** | **P** |  |
| VO_2_peak | 3.00 | 0.003 | 2.15  2.42  2.29  6.24  2.41  2.81 | 0.03  0.02  0.02  <0.001  0.02  0.005 | No difference – outcome remains significantly improved |
| Creatinine | 1.42 | 0.16 | 0.33  2.81  1.28  1.06 | 0.74  0.005  0.20  0.29 | **With Korabiewska et al. removed, we saw a reduction in creatinine following exercise** |
| Haemoglobin | 1.37 | 0.17 | 1.14  1.42  0.85 | 0.25  0.16  0.39 | No difference – exercise does not impact outcome |
| Systolic blood pressure | 0.28 | 0.78 | 0.33  0.53  0.31  0.06  0.58  0.12 | 0.74  0.60  0.75  0.95  0.56  0.90 | No difference – exercise does not impact outcome |
| Diastolic blood pressure | 0.16 | 0.87 | 0.66  0.55  0.23  0.38  0.60  0.19 | 0.51  0.58  0.82  0.70  0.55  0.85 | No difference – exercise does not impact outcome |
| HDL | 2.15 | 0.03 | 1.75  1.53  2.18 | 0.08  0.13  0.03 | **With Karelis et al. removed, exercise does not impact outcome** |
| LDL | 0.84 | 0.40 | 1.75  0.77 | 0.08  0.44 | No difference – exercise does not impact outcome |
| Triglycerides | 0.26 | 0.80 | 0.60  0.34 | 0.55  0.73 | No difference – exercise does not impact outcome |
| Fasting glucose | 0.10 | 0.92 | 0.00  0.10 | 1.00  0.92 | No difference – exercise does not impact outcome |
| Body mass | 1.17 | 0.24 | 1.06  1.35  0.39  1.20 | 0.29  0.18  0.70  0.23 | No difference – exercise does not impact outcome |
| BMI | 0.00 | 1.00 | 0.35  0.26  0.31  0.30 | 0.72  0.80  0.76  0.77 | No difference – exercise does not impact outcome |
| Maximum heart rate | 1.89 | 0.06 | 3.37  1.28  1.79 | <0.001  0.20  0.07 | **With Greenwood et al. removed, we saw an increase in heart rate following exercise** |
| Resting heart rate | 0.31 | 0.76 | 0.39  1.53 | 0.70  0.13 | No difference – exercise does not impact outcome |

Appendix TX8.

**Funnel plots**

|  |  |
| --- | --- |
| Creatinine | Fasting glucose |
|  |  |
| Hb | LDL |
|  |  |
| Triglycerides | Maximum heart rate |
|  |  |
| Resting heart rate | Systolic blood pressure |
|  |  |
| Diastolic blood pressure | Body mass |
|  |  |
| BMI |  |
